# *Trans*-regulatory changes underpin the evolution of the *Drosophila* immune response

**DOI:** 10.1371/journal.pgen.1010453

**Published:** 2022-11-07

**Authors:** Shuai Dominique Ding, Alexandre B. Leitão, Jonathan P. Day, Ramesh Arunkumar, Morgan Phillips, Shuyu Olivia Zhou, Francis M. Jiggins

**Affiliations:** 1 Department of Genetics, University of Cambridge, Cambridge, United Kingdom; 2 Champalimaud Foundation, Lisbon, Portugal; Friday Harbor Laboratories: University of Washington, UNITED STATES

## Abstract

When an animal is infected, the expression of a large suite of genes is changed, resulting in an immune response that can defend the host. Despite much evidence that the sequence of proteins in the immune system can evolve rapidly, the evolution of gene expression is comparatively poorly understood. We therefore investigated the transcriptional response to parasitoid wasp infection in *Drosophila simulans* and *D. sechellia*. Although these species are closely related, there has been a large scale divergence in the expression of immune-responsive genes in their two main immune tissues, the fat body and hemocytes. Many genes, including those encoding molecules that directly kill pathogens, have *cis* regulatory changes, frequently resulting in large differences in their expression in the two species. However, these changes in *cis* regulation overwhelmingly affected gene expression in immune-challenged and uninfected animals alike. Divergence in the response to infection was controlled in *trans*. We argue that altering *trans*-regulatory factors, such as signalling pathways or immune modulators, may allow natural selection to alter the expression of large numbers of immune-responsive genes in a coordinated fashion.

## Author summary

A fundamental question in biology is the nature of the genetic changes underlying evolutionary change, and immune systems provide an ideal system to examine this as they tend to evolve fast as animals adapt to an ever-changing array of parasites and pathogens. Comparing two species of the fruit fly *Drosophila*, we found that the transcriptional response to infection evolves extremely fast. However, changes in *cis* (where the genetic change is on the same DNA molecule as the gene in question) and *trans* (where the genetic change can be elsewhere in the genome) are playing different roles. Changes in *cis* frequently caused large differences in immune gene expression between species, but these differences were seen regardless of whether the animal was infected. In contrast, changes in *trans* were responsible for altering how gene expression changes in response to infection. Immune responses are complex and multifaceted, requiring the expression of many genes to be altered in a tightly regulated manner when the animal is infected. Natural selection acting on *trans* regulatory factors may allow the expression of many downstream genes to be altered in a coordinated fashion.

## Introduction

Pathogens and parasites drive rapid evolutionary change in their hosts, and it is common to find closely related species which differ greatly in their susceptibility to infection [1]. *Drosophila*, like most animals, relies on an innate immune system to defend itself against infection, where immune receptors and effectors are encoded in the germline. Infection is detected when receptors bind to non-self, such as peptidoglycan from bacterial cells or molecules derived from parasitic wasps [2]. Alongside this, the immune system can sense damage to host tissues, such as when a parasitic wasp ovipositor pierces the cuticle [3]. These processes activate signalling pathways, including the Toll and Imd pathways which culminate in NF-kappaB transcription factors translocating into the nucleus [4]. This results in a rapid transcriptional response [5]. Humoral immunity centres on the fat body, which secretes molecules such as antimicrobial peptides into circulation [4]. Meanwhile, immune cells called hemocytes (blood cells) proliferate, move into circulation, and differentiate into specialised forms [6]. These cells can phagocytize microbes, and produce the enzymes required for the synthesis of melanin, which can encapsulate pathogens and parasites. Together, these defences provide a powerful protection against infection.

Innate immune systems evolve rapidly. For example, in *Drosophila*, natural selection has driven the rapid divergence of the peptide sequence of immune-related genes [7–9]. However, despite changes in gene expression underpinning the immune response, it remains poorly understood how patterns of immune gene expression change during evolution. The expression of a specific immune effector such as an antimicrobial peptide could be changed in two ways: by altering *cis-*regulatory elements (CRE), which are DNA sequences that regulate the expression of nearby genes (e.g., promoters, enhancers), or upstream factors that control the expression of the gene in *trans*, such as pathogen recognition molecules, signalling pathways, transcription factors or immune modulators. A fundamental question is whether evolutionary change tends to occur when gene expression is altered in *cis* or *trans*. Work on a variety of species has found that *cis*-regulatory divergence between species is extremely common, affecting a large proportion of genes across genomes [10–14]. However, these *cis*-regulatory changes tend to be constant across environments, and differences in how gene expression responds to the environment are controlled in *trans* [13, 15, 16]. In this study we have investigated whether these principles also govern the evolution of gene regulation during the immune response.

In *Drosophila*, there is evidence that variation in both *cis*- and *trans*-acting factors controlling the expression of immune effectors may affect susceptibility to infection. Genetic polymorphisms in regulatory proteins, such as signalling pathway components, affect susceptibility [17], suggesting these may affect downstream effectors in *trans*. Furthermore, these interact with non-coding variants in antimicrobial peptide genes [17], suggesting a role for *cis* regulatory variation in effector genes. The role of *cis*- and *trans*-regulatory polymorphisms in controlling the expression of immunity genes in the fat body has been investigated in two lines of *D. melanogaster* that were infected with bacteria [18]. In uninfected flies, *cis*-regulatory polymorphisms were the dominant cause of differences in the expression of immune-responsive genes. This was also the case when flies were infected with Gram-positive *Enterococcus faecalis*, but when they were infected with Gram-negative *Serratia marcescens* differences in expression were mainly controlled in *trans* [18]. Together with its well-understood immune system, these observations mean *Drosophila* provides the opportunity to understand how regulation of genes expression changes during the evolution of immune systems.

To understand the changes to gene regulation that underlie the evolution of immune responses, we have compared two closely related species where an ecological shift is thought to have caused recent and rapid divergence in their immune defences. Parasitoid wasps are common parasites of *Drosophila*, laying their eggs in fly larvae and eventually killing their hosts. When a *Drosophila melanogaster* larva is parasitised, the fat body secretes humoral immune factors into circulation, while specialised hemocytes called lamellocytes differentiate [19]. If the immune response is successful, these cells encapsulate the parasitoid egg and undergo melanisation, killing the parasitoid [20]. The *Drosophila simulans* immune response against parasitoids closely resembles that seen in *D. melanogaster*. However, *Drosophila sechellia* is thought to have escaped from parasitoid attack by feeding on a fruit that is toxic to the wasps [21]. Following this ecological shift, it has evolved a greatly altered transcriptional response to parasitoid attack [22], produces few lamellocytes and is unable to encapsulate parasitoids [23, 24]. As these changes have occurred within the last few hundred thousand years [25], we have used these species as a model to understand the evolution of the transcriptional response to infection.

We used RNA sequencing to distinguish the *cis* and *trans* regulatory changes underlying the differing immune responses of these species. While differences in gene expression between the two species can be caused by both *cis* and *trans* divergence, in F1 hybrids alleles inherited from both parental species are within the same cellular environment and therefore under identical *trans* control. Therefore, the relative abundance of transcripts from the two alleles of a gene in F1 hybrids captures the *cis* regulatory differences between the two species [26, 27]. This means that by comparing the expression levels of the two alleles in hybrids (*cis*) to the total regulatory difference between parental species (*cis*+*trans*), we can estimate the extent of divergence in *trans* regulation [10, 14]. This approach has been applied to several closely related pairs of species, frequently with the result indicating that differences in gene expression are predominantly the result of *cis* differences [10–14]. Here, we have extended this approach to understand how *cis* and *trans* regulatory changes have led to the divergence of the immune responses of *D. simulans* and *D. sechellia*.

## Results

### *D. sechellia* has lost the ability to encapsulate

**Fig 1:**
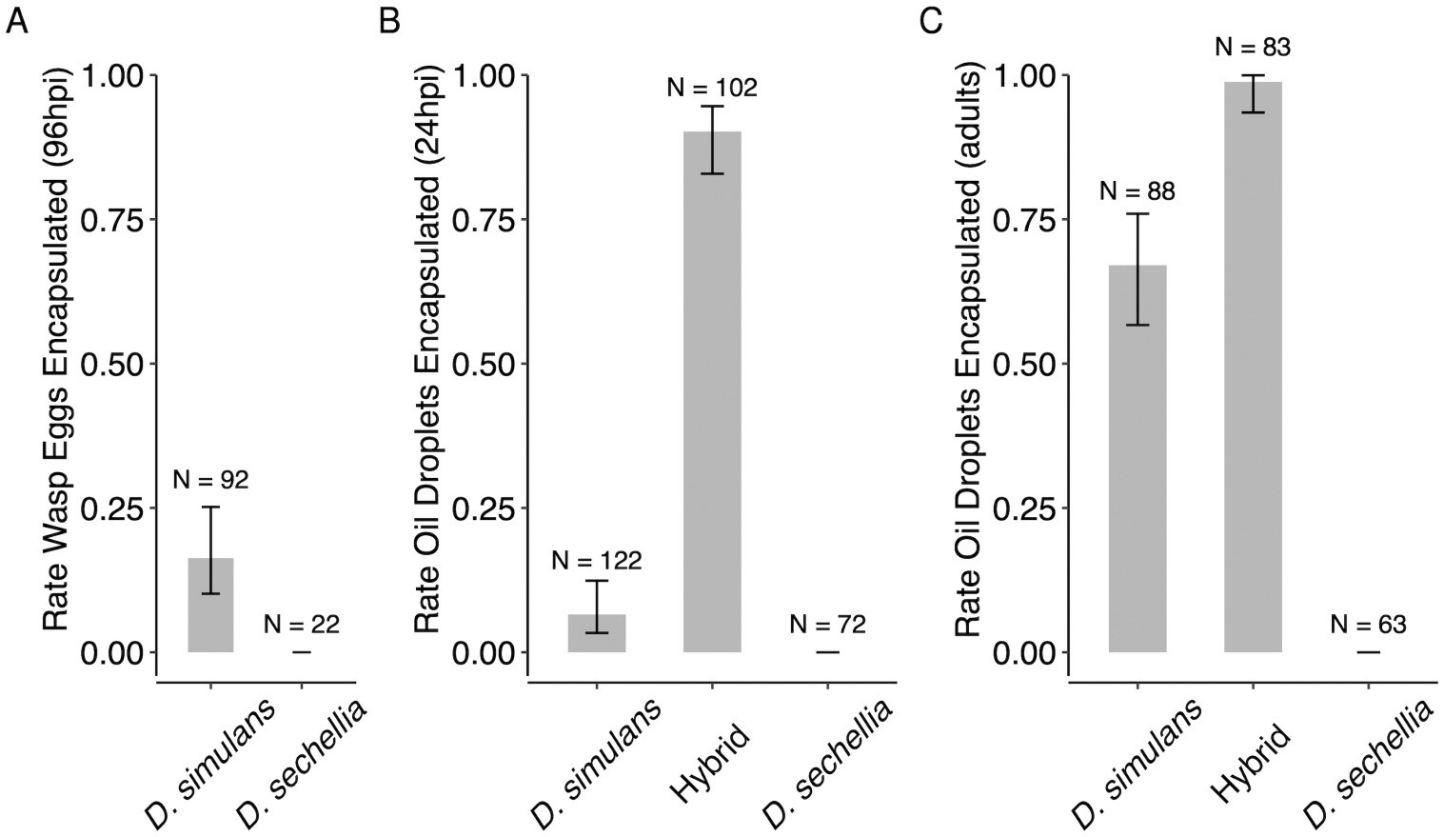
The immune response of two species of *Drosophila* and their hybrids. (A) The proportion of larvae that had encapsulated eggs of the parasitoid wasp *L. boulardi* 96 hours post infection (96hpi). (B and C). The proportion of encapsulated droplets of mineral oil containing wasp homogenate in (B) larvae 24 hours post injection or (C) in adult flies that developed from injected larva. Error bars are 95% binomial confidence intervals.

The *Drosophila* immune system kills parasitoid wasp eggs by encapsulating them in a layer of immune cells called hemocytes and then melanising this cellular capsule. To characterise how the susceptibility of *D. simulans* and *D. sechellia* to parasitoid wasp infection differed, we allowed *L. boulardi* to oviposit in the fly larvae and dissected them after 96 hours. In line with previous reports [23, 24], we found that *D. sechellia* never encapsulated the wasp eggs but *D. simulans* can mount an encapsulation response (Fig 1A).

Parasitoid wasps can suppress and evade the immune response. To investigate the immune response without any countermeasures from the parasitoid, we injected the larvae with a droplet of mineral oil that contains homogenised parasitoid tissue [2] and examined the rate the oil droplet was encapsulated (Fig 1). *D. simulans* had encapsulated 6.6% of oil droplets 24 hours post injection (hpi), which is a time-point when *Drosophila* larvae are encapsulating wasp eggs [28]. By the time adults developed from injected larvae, 67% of oil droplets were encapsulated. In contrast, *D. sechellia* is unable to mount an encapsulation response, as even when flies had developed into adults none of the oil droplets had been encapsulated. Unexpectedly, F1 hybrids encapsulated at higher rates than either parent, largely due to them mounting a faster response than *D. simulans*—90.2% of oil droplets were encapsulated in larvae and 98.8% in emerged flies (Fig 1).

### The cellular and humoral immune response of *D. simulans* and *D. sechellia* has extensively diverged

To understand the immunological reasons why the two species differ in susceptibility to infection, we examined the transcriptional response to immune challenge in the two main immune tissues of *Drosophila*—hemocytes and the fat body. We injected larvae with droplets of mineral oil containing wasp homogenate and measured gene expression by RNAseq 24 hours later. This time-point was selected as it is when parasitoids are being encapsulated [28], and there is a large transcriptional response to infection [29]. After filtering out low expression genes, there were 4969 and 5275 genes remaining in fat body and hemocyte data-sets respectively. Of these, 531 genes in fat body and 1971 genes in hemocytes exhibited significant (FDR < 0.05) expression changes after immune challenge in at least one species or F1 hybrids.

**Fig 2:**
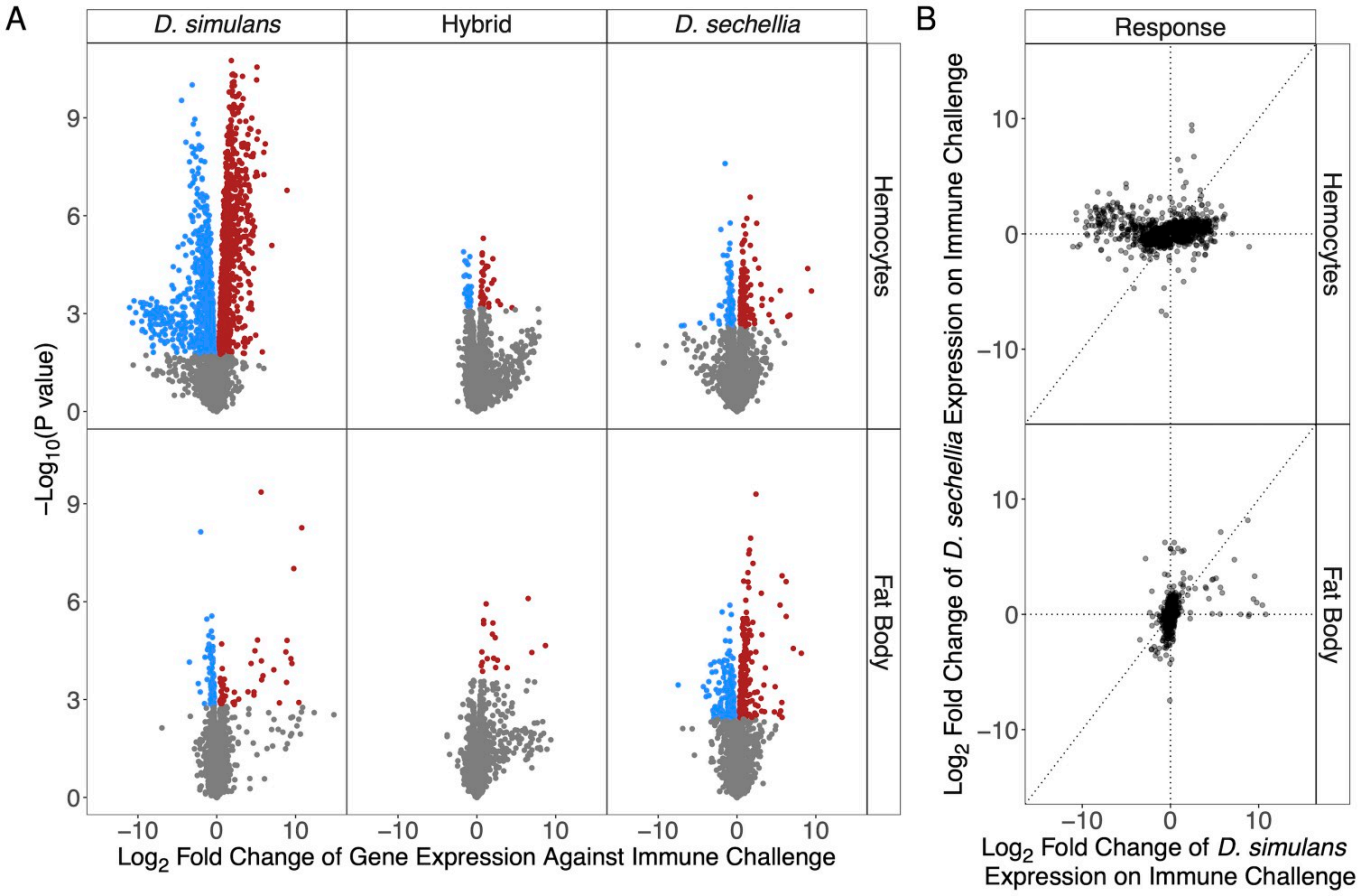
Divergence of the transcriptional response to immune challenge in fat body and hemocytes. Larvae were immune challenged by injecting them with mineral oil containing wasp homogenate while control larvae were not injected. The transcriptional response was then measured by RNA sequencing 24 hours post injection. (A) Volcano plots comparing the magnitude and statistical significance of changes in gene expression after immune challenge. Each point is a gene, and they are coloured above an FDR threshold of 5%. (B) The magnitude of the transcriptional response to immune challenge in *D. simulans* compared to *D. sechellia*. Each point is a gene.

In hemocytes, many more genes were differentially expressed in *D. simulans* than *D. sechellia* (FDR<0.05; coloured points in Fig 2A). Furthermore, different genes are changing their expression following infection in the two species, as there is only a weak correlation between their transcriptional response (Fig 2B; Pearson’s correlation of data in this figure: *ρ* = 0.06, *P*<0.007, 95% CI [0.02, 0.10]; S4 Dataset).

**Fig 3:**
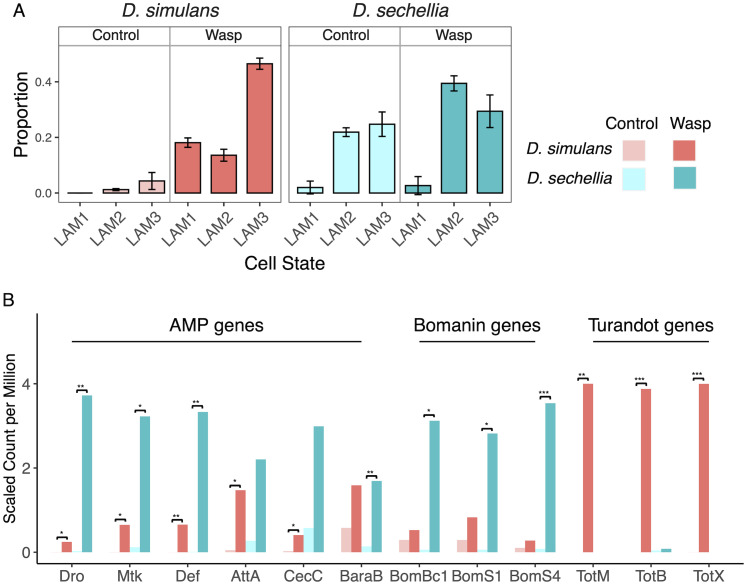
Changes in gene expression after immune challenge. (A) Proportion of lamellocytes in different transcriptional states in *D. simulans* and *D. sechellia* larvae under control and immune-challenged conditions, estimated from bulk RNA-seq data with CIBERSORTx. Error bars are 95% confidence intervals. (B) Changes in the expression of immunity genes in the *D. simulans* and *D. sechellia* fat body after immune challenge. Bars represent the scaled mean count per million of gene expression in control larvae and 24 hours after injection with mineral oil containing wasp homogenate. Only genes with a greater than two-fold change in expression in at least one species are shown. Significance of the change in expression after infection was defined by false discovery rate (FDR): 0.05*, 0.01**, 0.001***.

The cellular immune response to parasitoid wasps involves the differentiation of specialised immune cells called lamellocytes [30]. Using single-cell RNA sequencing, we have described how cells differentiate through immature forms (LAM1 and LAM2) to mature lamellocytes (LAM3) [31]. Using the data from that study, we used digital cytometry [32] to deconvolute our bulk RNA-seq data and infer the proportion of hemocytes in these different transcriptional states (Fig 3A). Despite *D. sechellia* producing few lamellocytes after infection, we unexpectedly inferred that mature and immature lamellocytes were present in uninfected *D. sechellia* larvae. The abundance of these cells only slightly increased after immune challenge. The presence of cells whose transcriptome resembles lamellocytes in *D. sechellia* is surprising as we know from studies of cell morphology that these cells are not produced in this species [23]. However, our previous work in *D. melanogaster* has shown that some genotypes of flies produce cells which transcriptionally resemble lamellocytes but only adopt the classical lamellocyte morphology after infection [31]. This suggests that the changes to the hemocyte population that have occurred in *D. sechellia* are more complex than simply losing the ability to differentiate lamellocytes.

In *D. simulans* there were few mature or immature lamellocytes before infection, but these cell states became abundant after immune challenge (Fig 3A). This contrasts with only small shifts in the cell population in *D.sechellia* Therefore, the larger transcriptional response of *D. simulans* hemocytes to immune challenge is likely caused by the differentiation of lamellocytes and their precursors.

In the fat body, there is weak correlation in the transcriptional response of the two species to immune challenge (Fig 2B; Pearson’s correlation in data in plot: *ρ* = 0.09, *P*<0.05, 95% CI [0.01, 0.17]; S4 Dataset), suggesting that the humoral immune response of the two species largely involves different genes. When looking at genes with large increases in expression after immune challenge, the difference between the two species is less stark than was the case for hemocytes, although the largest fold changes in expression were seen in *D. simulans* (Fig 2A).

The fat body plays a key role in the humoral immune response by secreting a diverse range of immune-related molecules into the hemolymph. We identified 84 differentially expressed genes with a log_2_ fold change greater than two in at least one species (FDR<0.05). As expected, a gene ontology term analysis found that these were enriched in genes involved in the defence response (*P*<0.05, Holm-Bonferroni corrected). A key role of the fat body is to secrete antimicrobial peptides (AMPs), and several of these were up-regulated in both species (Fig 3B). Bomanins are essential in killing some bacterial pathogens [33, 34], and they were significantly up-regulated upon immune challenge in *D. sechellia* but not *D. simulans* (Fig 3B). The opposite is the case for three genes encoding Turandots, which are stress response genes that are commonly induced after infection [35]. The expression of these genes increased approximately 1000-fold in *D. simulans*, but they were not significantly up-regulated in *D. sechellia* (Fig 3B). In most cases the differences in fold-change-after-infection between the species was not significant, but in the final section of the results we measure the expression of a selection of effector genes by quantitative PCR in whole larvae, and this confirmed that AMPs and Bomanins are more highly expressed in *D. sechellia* than *D. simulans*. Together, our results demonstrate that the humoral immune response of these closely related species to parasitoids has diverged considerably.

### *Trans-*regulatory factors underlie divergence in the cellular immune response

**Fig 4:**
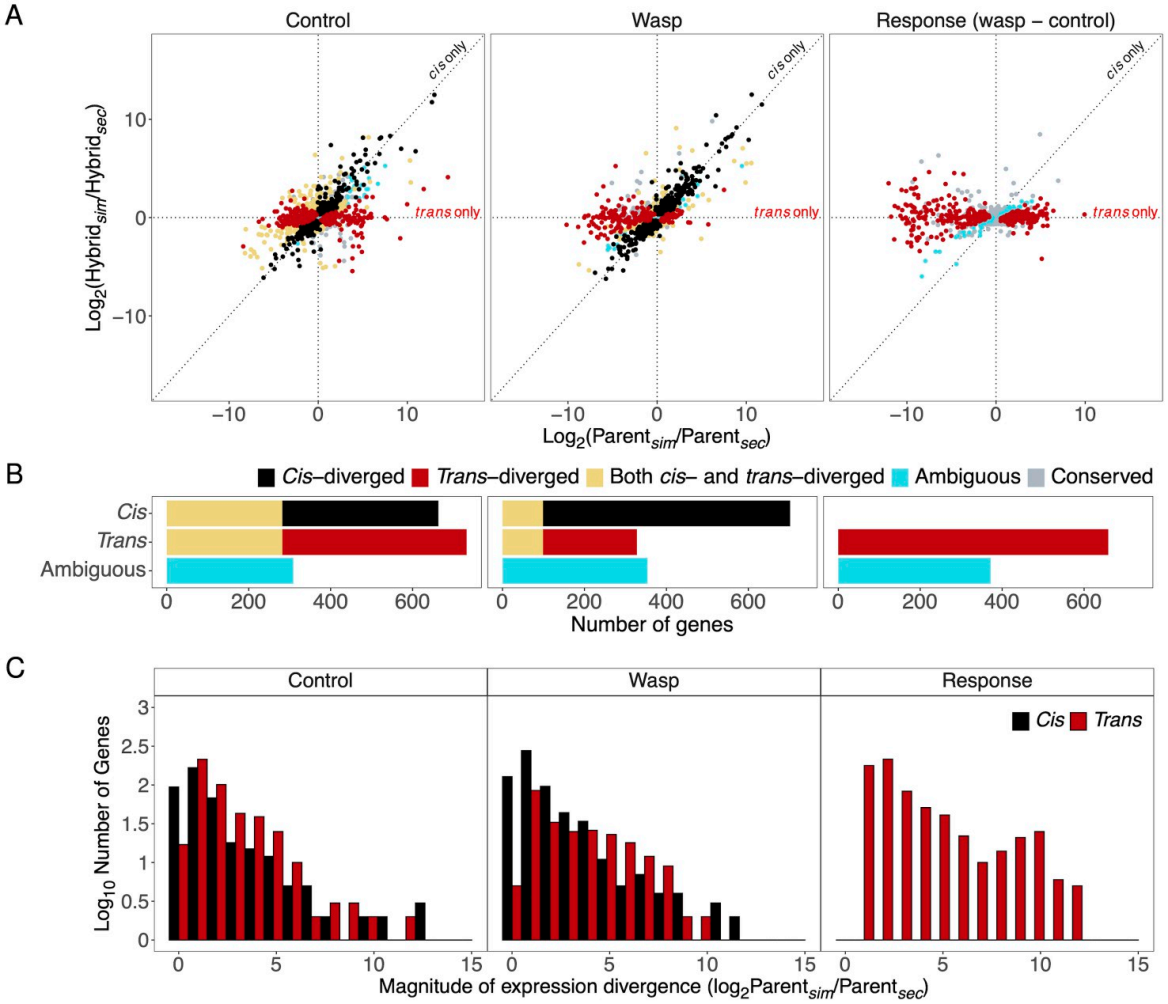
Expression divergence of immune responsive genes between *D. simulans* and *D. sechellia* in hemocytes. Plots only show genes that are significantly differentially expressed in response to infection in at least one species. (A) Expression differences between parental species (total regulatory divergence) plotted against allelic expression differences within F1 hybrids (*cis-*regulatory divergence). Gene expression was measured under unchallenged control conditions (left) and immune-challenged conditions (centre), and this was used to estimate the expression change in response to immune challenge (right). Each point is a gene, the expression difference of each gene is represented as the relative log_2_ fold change. (A and B) The genes are colour coded according to whether gene expression divergence is controlled in *cis* or in *trans*, with panel (B) depicting the number of genes in each category. The ambiguous category refers to genes with significant divergence in expression between the parental species but no significant divergence in either *cis* or *trans*. (C) Histograms depicting the frequencies of *cis-* and *trans-*diverged genes across the magnitudes of total expression divergence between *D. simulans* and *D. sechellia*. Fly larvae were immune-challenged by injecting them with oil droplets containing wasp homogenate.

Our data on allele-specific expression in hybrids allowed us to examine the relative importance of *cis* and *trans* regulatory changes in causing evolutionary divergence in the cellular immune response. Here, we restricted our analysis to the 1971 genes that were differentially regulated in hemocytes following immune challenge (Fig 4).

First, we examined the immune response—the magnitude by which expression changed following an immune challenge (Fig 4, right column). We found no evidence of any genes where the response to infection had diverged due to changes in *cis* (Fig 4A and 4B, right column; no genes where *cis* effect with FDR<0.05). In contrast, changes in *trans* altered the response to infection in 659 genes (Fig 4A and 4B, right column; FDR<0.05). Furthermore, the magnitude of these effects was frequently large, with changes in *trans* causing the response of some genes to differ by 1000-fold or more between *D. simulans* and *D. sechellia* (Fig 4C, right column). Therefore, differences in the cellular immune response of the two species are caused by *trans* regulatory divergence, likely controlling the differentiation of immune cells after infection (Fig 3A).

Next, we examined the expression of these immune-responsive genes under control conditions, where the immune response has not been activated. Here the pattern was dramatically different. As well as many immune-responsive genes with *trans-*regulatory divergence (732 of 1971 genes, 37.1%; FDR<0.05), there was also extensive *cis-*regulatory divergence (663 of 1971, 33.6%; FDR<0.05) (Fig 4A and 4B, left column). The divergence of both *cis* regulatory elements and *trans* regulatory factors was frequently associated with very large changes in gene expression, sometimes exceeding a 100-fold change in mRNA levels. However, there was a tendency for *trans* effects to cause larger changes in gene expression than *cis* effects (Fig 4C). To assess the overall importance of *cis* and *trans* effects, we examined the correlation between the total divergence in gene expression between the parental species and divergence in *cis* or *trans.* This revealed that total changes in gene expression were more strongly correlated with the *trans-*regulatory divergence (*ρ* = 0.73, 95% CI [0.71, 0.75], Pearson’s correlation of log_2_ fold change of gene expression across 1971 genes, S6 Dataset) than *cis-*regulatory divergence (*ρ* = 0.54, 95% CI [0.51, 0.57], S6 Dataset). A similar pattern emerges when looking at the expression of genes in the hemocytes of immune-challenged larvae. Here, the number of immune responsive genes that diverged under *trans-*control decreased (Fig 4B). However, the total gene expression divergence between *D. simulans* and *D. sechellia* remained strongly correlated with both *trans-* (*ρ* = 0.70, 95% CI [0.68, 0.73], S6 Dataset) and *cis-*divergence (*ρ* = 0.66, 95% CI [0.63, 0.68], S6 Dataset), due to the *trans-*regulatory changes leading to larger expression differences (Fig 4C). Overall, this data demonstrates that in both infected and uninfected larvae, divergence in the expression of immune responsive genes is caused by changes in both *cis* and *trans*. However, while changes in *cis* affect expression in both infected and uninfected larvae alike, changes in *trans* frequently alter how expression responds to infection.

### Dysregulation of cellular immunity in hybrids

To understand how regulatory factors of the two species interact, we examined the transcriptional response to immune challenge in hybrids. As the response in hemocytes is driven by the differentiation of lamellocytes, we initially examined the expression of *atilla*, which is specifically expressed in mature lamellocytes and commonly used to identify this cell type [36]. In uninfected F1 hybrids, the expression of *atilla* [36] was overdominant, exceeding the expression in *D. simulans* by 3.6 times and *D. sechellia* by 4.3 times. This is consistent with the immune response in hybrids becoming dysregulated, such that this cell type or its precursors are present even in uninfected individuals. After immune-challenge, the expression of *atilla* increased in *D. simulans* but not *D. sechellia*. This resulted in *atilla* expression in *D. simulans* and hybrids being respectively 32 and 16 times that in *D. sechellia* (*p* < 10^−6^, quasi-likelihood *F*-test). This is consistent with the observation that few lamellocytes differentiate in *D. sechellia*.

These observations indicate that hybrids already possess activated hemocytes pre-infection, and the immune challenge in *D. simulans* causes it to ‘catch up’ with hybrids. To explore this further, we used digital cytometry (see above) to estimate the proportion of hemocytes in different transcriptional states in hybrids. As was the case in *D. sechellia*, lamellocytes or their precursors were present before immune challenge. However, after immune challenge the number of these cells increase (S5 Fig). Therefore the hybrids combine features of both parents—activation of these cells before infection (*D. sechellia*) and induction after infection (*D. simulans*). As a consequence of these cell states being present before immune challenge, the magnitude of the transcriptional response to infection in hybrid larvae was far less than in *D. simulans* (Fig 2A).

There was also a substantial number of other genes that were misexpressed in the hemocytes of hybrids, with expression levels sometimes deviating strongly from levels in both the parental species (S4 Fig). In unchallenged larvae, this was apparent in a group of genes that had lower expression in the hybrids than either parent, a pattern known as underdominance (S4(A) Fig, left column). After immune challenge, the expression of many of these genes was restored to normal levels (S4(A) Fig, centre column). This increase in gene expression after infection means that their response to immune challenge was overdominant (S4(B) Fig, right column). Other genes showed dominance of the *D. sechellia* allele resulting from the expression of lamellocyte markers described above (S4(B) Fig).

### *Trans-*regulatory changes drive divergence in the humoral immune response

**Fig 5:**
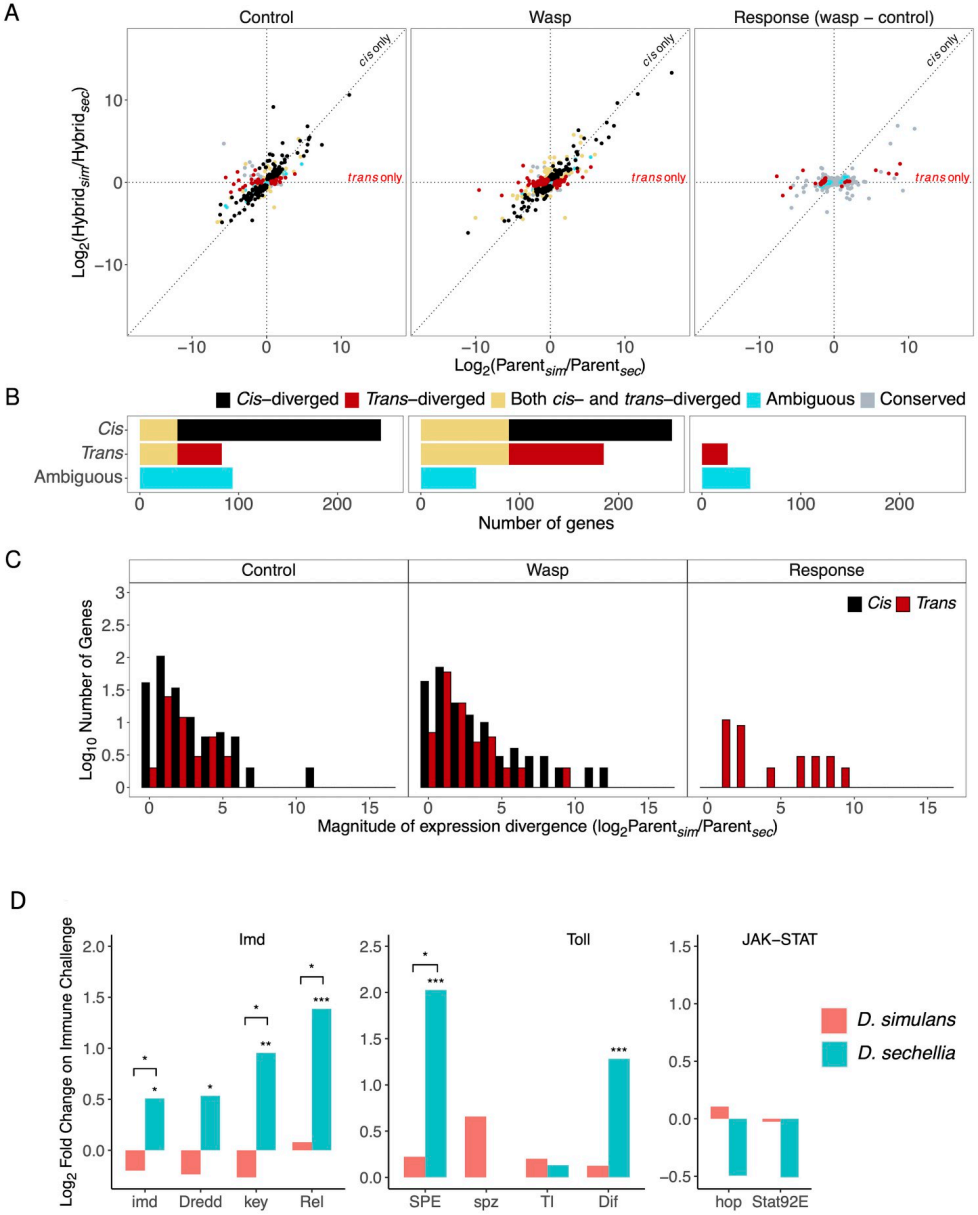
Expression divergence of immune responsive genes between *D. simulans* and *D. sechellia* in the fat body. Plots only show genes that are significantly differentially expressed in response to infection in at least one species. (A) Expression differences between parental species plotted against allelic expression differences within F1 hybrids (*cis-*regulatory divergence). Gene expression was measured under unchallenged control conditions (left) and immune-challenged conditions (centre), and this was used to estimate the expression change in response to immune challenge (right). (A and B) The genes are colour coded according to whether gene expression divergence is controlled in *cis* or in *trans*, with panel (B) depicting the number of genes in each category. (C) Histograms depicting the frequencies of *cis-* and *trans-*diverged genes with differing expression divergence between *D. simulans* and *D. sechellia*. (D) Changes in the expression of immunity genes encoding components of major humoral immune response signalling pathways in the fat body after immune challenge. Bars represent the change in gene expression 24 hours after injection with mineral oil containing wasp homogenate. Significantly differentially expressed genes within each species were indicated by * on top of columns. Divergent gene expression change responding to immune challenge between *D. simulans* and *D. sechellia* was indicated by * on top of bars between columns. Significance level was defined by false discovery rate (FDR): 0.05*, 0.01**, 0.001***.

Unlike hemocytes, the humoral immune response in the fat body is not known to involve the differentiation of new cell types, but instead leads to cells secreting molecules into the haemolymph. As was the case for hemocytes, we focused our analysis on genes that were differentially expressed in at least one of the species after immune challenge. In our control flies where the immune response has not been activated, the expression of 46% of these genes had diverged in *cis* (244 of 531 immune responsive genes; Fig 5A and 5B). In contrast, only 15.6% of the genes had significant divergence in *trans* (83 genes; Fig 5A and 5B). As a consequence, the total divergence of gene expression between the two species was more strongly correlated with *cis-*regulatory divergence than *trans-*regulatory divergence (*cis*: Pearson’s *ρ* = 0.73, 95% CI [0.69, 0.77]; *trans*: *ρ* = 0.55, 95% CI [0.49, 0.61]). Therefore, in uninfected larvae, differences in the expression of immune-responsive genes are predominantly caused by changes to *cis* regulatory elements.

In contrast to the situation in uninfected larvae, divergence in the response to immune-challenge between the two species is driven by changes to *trans* regulatory factors. Examining the fold-change in gene expression after immune activation, we did not find any genes where there were significant *cis* regulatory differences, but 31 genes had significant divergence in *trans* (Fig 5A and 5B, right-hand column). The importance of *trans* effects is reflected in a strong correlation between the divergence in the response of these genes to immune-challenge and the *trans-*effect (Fig 5) (*ρ* = 0.93, 95% CI [0.91, 0.94], Pearson’s correlation).

These analyses demonstrate that while *cis* regulatory divergence is the dominant factor causing differences in the expression of humoral immune genes in uninfected flies, changes in the response to infection is controlled in *trans*. The combination of these two effects is apparent when comparing levels of gene expression in immune-challenged larva of the two species. Here, the number of immune-responsive genes with *trans-*regulatory divergence has increased compared to unchallenged control conditions (Fig 5B, centre plot), as has the correlation between the divergence in gene expression and *trans-*regulatory divergence (*ρ* = 0.65, 95% CI [0.60, 0.70], Pearson’s correlation).

The humoral immune response in the fat body is regulated in a large part by the immune deficiency (Imd), Toll and JAK-STAT pathways, often acting in combination. For example, *Baramicin A* and *Bomanin* genes are regulated by the Toll pathway [37–39], while *Cecropin A*, *Attacin A*, *Defensin* and *Metchnikowin* are regulated to varying degrees by both Toll and Imd. Therefore, differences in the expression of Bomanin and AMP genes between *D. sechellia* and *D. simulans* suggest the Toll pathway and potentially other immune signalling pathways may be causing differences in expression in *trans* (Fig 3B, see below for verification by quantitative PCR). We therefore investigated whether genes encoding components of immune signalling pathways differed in their expression between the species. We found that six genes in the Toll and Imd pathways, including those encoding the NF-*κ*B transcription factors Dif and Relish, were significantly up-regulated upon immune challenge in *D. sechellia* but not *D. simulans* (Fig 5D). In four of these cases the difference in immune induction was significant between the species (Fig 5D). The up-regulation of these genes in *D. sechellia* therefore provides a potential explanation of why Bomanins and AMPs are more highly expressed in this species.

To examine whether these pathways might underlie differences in gene expression between the species that are regulated in *trans* more broadly, we searched for binding site motifs of six immune related transcription factors (Relish, Dif, Dorsal, STAT, Serpent and CrebA) in the region 1kB upstream of the 531 immune responsive genes. However, we failed to find any enrichment of these motifs upstream of genes showing *trans* regulatory divergence (S2A Table). Similarly, there was no association between genes where only one species possessed the motif and divergence in *cis* (S2B Table).

To further understand how *trans*-regulatory factors had diverged between these species, we examined the immune response in F1 hybrid larvae. In contrast to hemocytes, the immune response of the hybrid fat body most closely resembled *D. simulans*. In unchallenged control fat bodies, there was no clear tendency for gene expression in hybrids to resemble either parent (S4 Fig). However, after infection the level of expression in *D. simulans* tended to be dominant, with changes in expression level in the hybrids being similar to *D. simulans* (S4(B) Fig). This is consistent with a dominant *trans*-acting factor inherited from *D. simulans* controlling the humoral immune response in hybrids.

### *Cis-*regulatory divergence causes large changes in the expression of immune effectors

**Fig 6:**
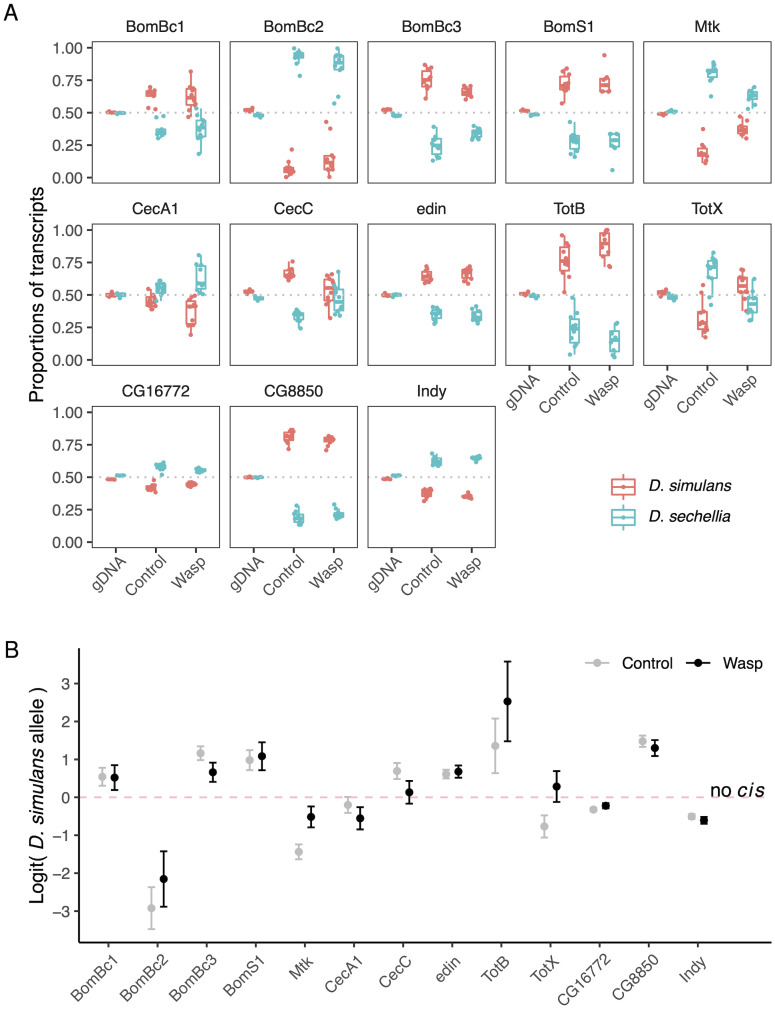
*Cis* regulatory divergence in humoral immunity genes. Transcripts were amplified by PCR from F1 hybrids between *D. simulans* and *D. sechellia*, and the PCR products Illumina sequenced. RNA was extracted from whole larvae that were uninfected (control) or immune challenged (wasp). Genomic DNA (gDNA) was included to check there were no biases towards one allele. (A) The proportion of reads from the two alleles. (B) The natural log of the odds (logit) that a transcript is from the *D. simulans* allele. Error bars are 95% confidence intervals on model coefficients. Fly larvae were immune-challenged by injecting them with oil droplets containing wasp homogenate. The horizontal dashed line (*y* = 0.5) signifies that there is no *cis-*regulatory divergence between the two species.

To provide increased statistical power to examine how changes to *cis* regulatory elements affect the transcriptional response to infection, we investigated divergence in the expression of a small number of humoral immune-response genes with greater replication. As reported previously, parasitoid exposure up-regulates antimicrobial peptides and stress response genes [29]. As many of these genes had considerable *cis* differences between species, we selected 13 genes that were mostly known to play a role in these responses. To allow us to increase our sample size, we used whole larvae rather than dissecting single tissues, so the results are not directly comparable to the RNAseq experiment. We designed primers to amplify targets within exons of these genes from cDNA or genomic DNA templates and sequenced the products using Illumina sequencing (S1 Table). We used SNPs within the reads to determine whether they were from the *D. simulans* or *D. sechellia* allele. When we amplified genomic DNA from F1 hybrids, approximately half the reads were from the *D. simulans* allele and half from the *D. sechellia* allele, confirming there is no PCR bias causing one of the alleles to be preferentially amplified (Fig 6A).

There was allele-specific expression of all 13 genes in F1 hybrids, indicating that there is interspecific *cis-*regulatory divergence (Fig 6A and 6B). In some cases, the magnitude of these effects is large. For example, for *Bomanin Bc2* (*BomBc2*) there were 18 times more *D. sechellia* transcripts than *D. simulans* transcripts in unchallenged larvae. Similarly, after immune challenge, there was more than 12 times more *D. simulans* *TotB* transcripts than *D. sechellia* transcripts (Fig 6B). These *cis-*regulatory differences affected several classes of immune effectors where the concentration of the gene product is expected to affect susceptibility to infection. For example, Bomanins are a family of secreted proteins induced after infection that play a central role in killing Gram-positive bacteria and fungi [33, 34]. We found that the *D. simulans* allele of three *Bomanin* genes had greater expression, but in the fourth gene the *D. sechellia* allele was more highly expressed (Fig 6A and 6B). The antimicrobial peptides Metchnikowin (*Mtk*) and Cecropin (*Cec*) kill fungi and Gram-negative bacteria respectively. *Mtk, CecA1* and *CecC* all showed evidence of allele-specific expression, and again in some cases the *D. simulans* allele was more highly expressed, and in other cases the *D. sechellia* allele (Fig 6A and 6B). Similarly, we found that there was significant *cis-*regulatory divergence in the expression of the parasitoid-induced gene *Edin* [40] in both uninfected and immune-challenged larvae.

### *Cis-* and *trans*-regulatory changes combine to switch *TotX* between inducible and constitutive expression

**Fig 7:**
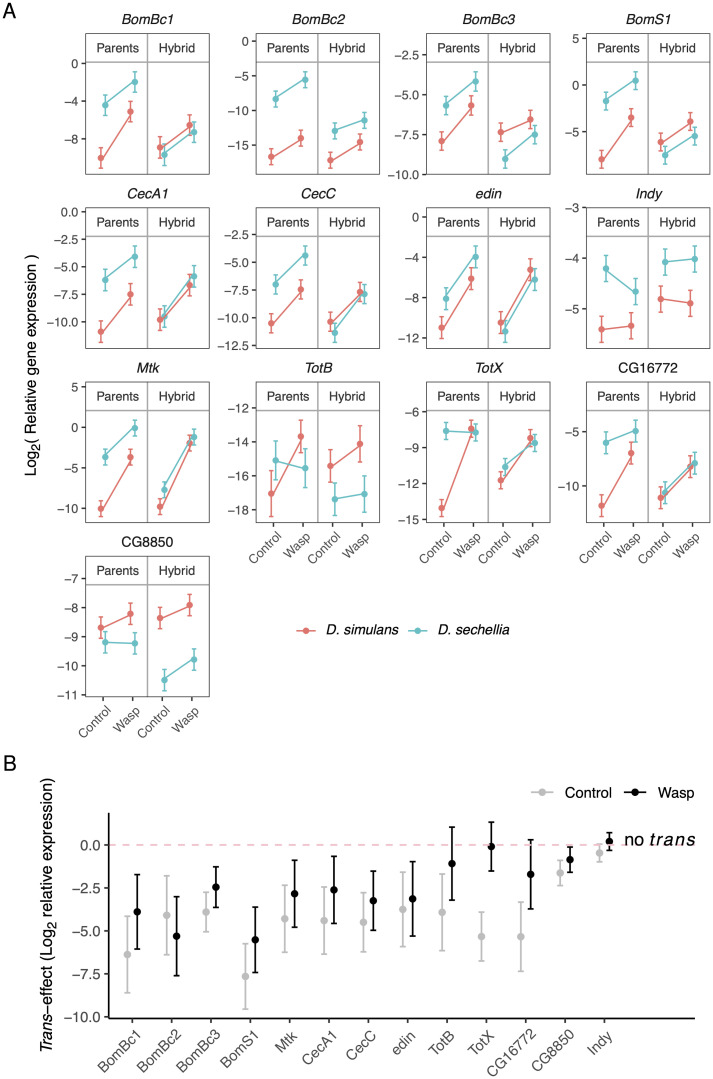
Relative expression between *D. simulans* and *D. sechellia* alleles and *Trans* regulatory divergence in humoral immunity genes. Relative gene expressions were quantified by qPCR in parental and F1 hybrid larvae. The relative allelic expression in hybrids were estimated using proportions inferred from Illumina MiSeq sequencing of hybrid transcriptions for each gene, and the relative allelic expression between parental larvae were calibrated to half accordingly. (A) Relative allelic expressions between parental larvae and F1 hybrids under unchallenged (control), and infected (immune challenge imposed by injections of wasp homogenate) conditions. (B) *Trans-*regulatory divergence between *D. simulans* and *D. sechellia* in unchallenged and infected larvae, measured as the log_2_ ratio of relative *D. simulans* over *D. sechellia* expression. Error bars are 95% confidence intervals on model coefficient.

To investigate how the expression of these 13 humoral response genes differed between the parental species, we measured their expression by quantitative PCR. This revealed that the stress-response gene *TotX* is up-regulated ∼100X after immune challenge in *D. simulans*, but it is constitutively expressed at a high level in *D. sechellia*, irrespective of whether the immune response has been activated (Fig 7A). To understand the role of *cis* and *trans* divergence in this evolutionary shift, we also measured the expression of this gene in the F1 hybrids. By combining these measurements with the allele-specific expression data described above (Fig 6), we could estimate the expression level of each allele in hybrids (Fig 7A). This revealed that both the *D. simulans* and *D. sechellia* alleles are immune-induced in F1 hybrids, demonstrating that *trans* regulatory differences between the species are essential for the gene to be constitutively expressed in *D. sechellia* (Fig 7B; interaction of the parameters allele, Parents/Hybrids and immune challenge, i.e., *trans* regulatory effect on immune response: *p* < 10^−5^ after Bonferroni correction). Using this data to estimate the magnitude of *trans*-regulatory divergence between species, we found *trans*-acting factors cause large *TotX* expression differences in uninfected larvae of the two species (Fig 7B, grey points). In contrast, there was no significant *trans*-regulatory divergence in infected larvae (Fig 7B, black points).

While divergence in *trans* is required for *TotX* to switch between constitutive and induced expression, *TotX* was also the only gene where the direction of allele-specific expression in F1 hybrids was reversed when flies were immune challenged (Fig 6). The *D. sechellia* allele is more highly expressed in uninfected larvae, but the *D. simulans* allele is most highly expressed after immune challenge (Fig 6; GLM: *p* < 10^−5^ after Bonferroni correction). Together, this suggests that the evolutionary transition between this gene being immune-induced and constitutively expressed likely results from the interaction between changes in both *cis* and *trans*.

We were able to identify this *cis*-regulatory change in *TotX* that altered the immune response due to the greater statistical power of this dataset compared to the RNAseq data. We therefore repeated this analysis on the 12 other selected genes and found that there were three where the degree of allele-specific expression differed between uninfected and immune-challenged larvae (Fig 6; *Mtk, BomBc3, CecC*; GLM: *p* < 0.01 after Bonferroni correction). In the case of *Metchnikowin*, an antifungal peptide, the magnitude of allele-specific expression is reduced when flies are immune-challenged (Fig 6; GLM: *p* < 10^−9^ after Bonferroni correction). However, the other two genes had only subtle changes in allele-specific expression after infection. While these results demonstrate that *cis*-regultory changes can affect the response to infection, this sample of genes were hand-picked based on the RNAseq data, so it does not challenge the general conclusion from the unbiased RNAseq analysis that most divergence in the immune response is controlled in *trans*.

We also measured the expression of these genes by quantitative PCR and estimated the *trans*-regulatory divergence in the same way as for *TotX*. All but *Indy* showed significant *trans* regulatory divergence in unchallenged larvae, and this remained significant in eight of the genes post immune challenge (Fig 7B). It is striking that all these genes also had significant *cis*-regulatory divergence (Fig 6), supporting our RNAseq analysis where many genes were affected by both *cis* and *trans* divergence. The interplay between these changes is illustrated by the *Bomanin* genes, which encode peptides that kill bacteria and fungi. All four of these genes were most highly expressed in *D. sechellia*, and *trans* regulatory divergence increased the expression of all four genes in this species (Fig 7A). However, in three of the four genes, *cis* regulatory divergence had the opposite effect, with the *D. sechellia* allele being most highly expressed (Fig 7A). This effect is known as ‘compensation’, and may evolve if stabilising selection acts on CREs to restore gene expression levels to their original level after a *trans*-regulatory element has changed. In the fourth gene, *BomBc2*, the *cis* and *trans* effects reinforced each other (Fig 7A).

## Discussion

Following an immune challenge, *D. simulans* and *D. sechellia* both exhibited large transcriptional responses, with hundreds of genes changing their expression level across the two major immune tissues, hemocytes and the fat body. However, despite these species being closely related, the nature of this transcriptional response has diverged considerably, resulting in there being little correlation between the responses of the two species. As the genes involved include key immune effector molecules and markers of immune cell differentiation, these transcriptional differences likely underpin the differences in susceptibility to infection seen between these two species. The protein sequence of *Drosophila* immunity genes frequently evolves rapidly [7], and these results demonstrate that the same is true for gene expression. Divergence in both *cis*- and *trans*-regulatory factors have caused large changes in the expression of many immune-responsive genes. However, changes in *cis* overwhelmingly altered constitutive levels of gene expression, affecting immune-challenged and uninfected animals alike. In contrast, changes in the response to infection were caused by *trans*-regulatory factors (Figs 4 and 5).

An important question is which pathways are causing divergence in *trans*. We found that a number of immune effector genes are more highly expressed in *D. sechellia* due to *trans*-regulatory divergence. A possible explanation is that genes encoding transcription factors and signalling pathway components have diverged in their expression between the two species, with components of both the Toll and Imd pathways being significantly up-regulated after infection in *D. sechellia* but not *D. simulans*. This result is reminiscent of a study of two lines of *D. melanogaster* where *trans* regulatory polymorphisms had affected the immune response to a Gram negative bacterium [18]. These lines differed in the expression of several regulators of the Imd pathway, which controls the response to Gram-negative bacteria [18]. However, when we predicted whether genes were regulated by the Toll or Imd pathways from the presence of transcription factor binding motifs, we were unable to link divergence in *trans* with the presence of specific motifs. We would caution however that we do not now how accurately this simple analysis predicted immune-responsive enhancers.

Immune responses are complex, requiring many genes to be differentially expressed in a coordinated fashion. Altering *trans*-regulatory factors may provide a mechanism by which natural selection can alter these complex responses in a coordinated fashion that would be difficult to achieve by altering the CREs of numerous effector genes. Our data cannot identify what these *trans*-acting factors are, but they may be involved in recognising infection, signalling or immune modulation. The nature of the changes to these upstream genes is also unknown—they could have changed in *cis*, *trans*, sequence, or been lost or duplicated.

The patterns we see may be an example of a more general phenomenon, with *trans*-regulatory changes underpinning the evolution of how gene expression responds to the environment [15]. For example, comparing yeast species across different media, *cis* divergence remains consistent across environments, while *trans* divergence differed between environments [13]. Similarly, in *Caenorhabditis elegans*, *trans* eQTLs were responsible for differing responses to heat stress while *cis* eQTLs were unspecific and shared across environments [16]. In the *D. melanogaster* fat body, polymorphisms in gene expression are mostly controlled in *cis* in uninfected flies, but in *trans* after *S. marcescens* infection (although not after *E. faecalis* infection). These studies mirror our observations, where we found the *trans* regulatory divergence driving the divergence of immune response, while changes in *cis* were constant across infected and uninfected animals. *Trans*-regulatory changes to gene expression may be environment-dependent because *trans*-regulatory pathways have evolved to control an organism’s plastic phenotypic response to the environment, or because mutations that alter the environmental sensitivity of CREs rarely arise.

The relative roles of *cis* and *trans* changes in evolution have long been debated by developmental biologists, who have argued that CREs play an important role in the evolution, in part because their modular structure allows them to alter expression of a single gene in specific cells at specific times [41]. In contrast, changes in *trans* may affect many downstream genes, causing harmful pleiotropic side effects [41]. These arguments apply to developmental patterning genes that regulate the expression of many other genes, while we are mainly studying terminal effector genes where expression changes are predicted to have fewer pleiotropic effects [42]. In this regard, immune systems may resemble other systems controlling organisms’ response to environmental stressors, such as heat shock, which tend to have many terminal effector genes and fewer regulatory genes [43]. These systems are thought to be more resilient to *trans* changes, due to less interconnection between *trans*-regulatory pathways [43].

While differences in the response to infection are overwhelmingly controlled in *trans*, we did find a few genes where changes in *cis* alter the response to infection. Over longer periods of evolutionary time, the accumulation of these *cis* changes may allow the nature of immune responses to change by altering the expression of specific genes. Our clearest example of *cis* changes altering the response to infection was in the stress response gene *TotX*, where the direction of allele-specific expression switched upon infection. However, in this gene there was also massive divergence in *trans*, and it is likely the interaction of these *cis* and *trans* changes that switched the gene between immune-induced and constitutive expression.

We activated the anti-parasitoid immune response, and parasitoids are known to drive especially rapid evolution of their hosts [23, 44]. The immune response of these species is thought to have rapidly diverged following an ecological shift where *D. sechellia* escaped from the parasitoids following a change in the fruit on which it feeds [21]. Therefore, the selection pressures driving this change likely reflect a relaxation of selective pressure, as opposed to directional selection as may commonly be the case during the coevolution of hosts and parasites. It therefore remains to be seen whether the patterns we observe can be generalised to immune system evolution more broadly. Nonetheless, such relaxation of selection may be common, as pathogens come and go during evolution. Furthermore, many of the genes that were differentially expressed are thought to be involved in killing microbes and not parasitoids. This broad immune response following parasitoid infection has been observed in other *Drosophila* spp., where there is strong up-regulation of antimicrobial peptides [1, 22, 29, 45]. These genes are likely up-regulated because our immune challenge (like parasitism) injures the fly larvae [3, 46], and because the same signalling molecules are used in different aspects of the immune response. Notably, it was recently found that extracellular serine proteases that regulate the Toll pathway also regulate the melanisation response [47]. Therefore, the divergence of the anti-parasitoid immune response and the Toll-regulated genes like Bomanins may be caused by the same molecular changes and selection pressures. Regardless of its causes, it gave us insights into the evolution of immunity beyond just factors involved in killing parasitoids.

There is a fundamental difference between the causes of changes in gene expression in the cellular and humoral immune responses. The cellular immune response relies on the differentiation of new cell types [48–50], and many of the changes we see likely result from the differentiation of specialised immune cells called lamellocytes in *D. simulans*. This can explain why there is far greater differential gene expression following immune challenge in *D. simulans* than *D. sechellia*. In contrast to our results in hemocytes, the humoral response in the fat body does not involve the differentiation of new cell types, and here *trans* regulatory factors are more likely to be affecting classical immune signalling pathways controlling the immune response. Despite distinctive characteristics of humoral and cellular immunity, there is extensive cross-talk between them during immune activation. Hemocytes mediate signals to the fat body during bacterial infection, facilitating in the activation of immune pathways and induction of antimicrobial peptides released by the fat body [51, 52]. On the other hand, activation of the Toll pathway in the *Drosophila* larval fat body is sufficient to launch a cellular response parallel to that after parasitoid oviposition, albeit inconsequential in defence against the parasitoid wasp, *Leptopilina boulardi* [53]. These interactions are likely underlying many of the patterns we see, with changes in the hemocytes causing changes in gene expression in the fat body or vice versa.

In conclusion, we have found that *cis*- and *trans*-regulatory changes play fundamentally different roles during the evolution of *Drosophila* immunity. Changes to the response to infection almost exclusively result from changes to *trans*-regulatory factors, while *cis* regulatory elements alter the expression of specific genes in both infected and uninfected animals.

## Materials and methods

### *Drosophila* lines and larvae preparation

The Sz49 line from the *D. simulans* panel [54] was selected as one of the parental lines. They were first collected in Zuma beach, California in 2012 and went through 15 generations of full sib matings from a single-mated female. We confirmed they were capable of encapsulating *Leptopilina boulardi* (strain *G486*) eggs prior to the experiment. The *D. sechellia* line DenisNF100 was from an isofemale line first collected from Denis Island in the Seychelles in 2012 [55], and subsequently sequenced [56].

*D. simulans* (Sz49) [54] and *D. sechellia* (DenisNF100) [56] were reared at 25°C with a 12-hour light/12-hour dark cycle. Virgin female flies were collected within 4 hours of emergence from the pupa and kept in vials for 2 days to confirm they did not lay fertile eggs. Mesh netting was placed over 50 mm Petri dishes containing standard cornmeal *Drosophila* food [57] with flies to limit space and encourage mating. To further encourage cross-species mating, twenty *D. simulans* virgin females were crossed with forty *D. sechellia* males in each Petri dish to produce F1 hybrids. Twenty *D. simulans* of each sex were used in each Petri dish for larvae production. To account for low egg production in *D. sechellia*, thirty flies of either sex were used in each Petri dish for larvae production. Ten Petri dishes were set up for each species and F1 hybrid.

Second instar female larvae were collected from each species and the hybrids 48 hours after hatching and assigned either to control groups with no treatment, or to being injected with wasp homogenate to induce an immune reaction. To produce the wasp homogenate, twenty male *L. boulardi* were placed on ice and homogenised in 200 μL of mineral oil with plastic pestles. For immune inductions, 4.6 nL of wasp homogenate was injected using a Nanoject II micro-injector (Drummond scientific, Bromall, PA, USA) into each larva towards the posterior end.

### Calculating encapsulation rate

To quantify the encapsulation rates of *D. simulans* and *D. sechellia* against wasp eggs, twenty second instar larvae were collected from each species and put in a cornmeal vial with 2 female *L. boulardi* for 3 hours in 18°C. After oviposition, the wasps were taken out of the vials and the infected fly larvae were left in 25°C for 96 hours before encapsulation assay. At 96 hours post infection (hpi), infected larvae were collected and washed in double-distilled water. Oviposition was confirmed by dissecting the infected larvae with tweezers under microscope to reveal either a wasp larva or a melanised egg. The encapsulation rate was calculated by dividing the total number of the fly larvae with a confirmed wasp oviposition by the number of fly larvae with an encapsulated wasp egg. For encapsulation assay in fly larvae against wasp homogenate, injected larvae were collected at 24 hpi and washed in double-distilled water before inspection for capsules by eye under microscope. For encapsulation assay in adult flies against wasp homogenate, the flies emerged from injected larvae were collected then anaesthetised by carbon dioxide and squashed between 2 microscope slides before placed under the microscope for visual inspection for capsules. Encapsulation rate was calculated as dividing the total number of larvae or fly by the number of larvae or fly that, through visual assessment, encapsulated the wasp homogenate droplet.

### Library preparation for RNA sequencing

At 24 hpi, groups of 70 larvae were bled for hemocytes by tearing the cuticle from the ventral side with tweezers, and groups of 10 larvae were dissected for fat bodies for each tissue-specific sample. Five replicates of fat body tissue were created for each species in injected and control groups, and three replicates for hemocyte tissue. RNA was isolated from 3rd instar control or infected larval tissues. For each sample, either fat body from 8 whole larvae or hemocytes from 70 larvae were homogenised in 500 μL Tri-reagent (Ambion 10296010) by pipetting up and down thoroughly with a P1000 pipette. 100 μL of chloroform was added, tubes shaken vigorously for 3 minutes then centrifuged for 10 minutes at 12,000g for 10 minutes. 150 μL of the upper aqueous phase was removed to a fresh tube and 2.5x volumes of propan-2-ol was added and mixed by inverting the tubes several times. After an incubation of 10 minutes at room temperature tubes were centrifuged at 12,000g at 4°C for 10 minutes, the supernatant carefully removed by pipetting and 250 μL ice-cold ethanol was added. The tubes were centrifuged for 2 minutes at 12,000g and 4°C and the ethanol was removed, the RNA pellets were air dried briefly and 30 μL nuclease free water was added and fully dissolved by incubating tubes at 45°C for 5 minutes. RNA was quantified using a Qubit RNA HS assay kit (Thermofisher Q32852). RNAseq libraries were prepared using an Ultra II stranded mRNA library kit (New England Biolabs E7760) with NEB Next multiplex unique indices (New England Biolabs E6440S) and polyA enrichment module (NEB E7490S). Up to 1 μg of total RNA was used to make each library. After polyA enrichment with the polyA enrichment module according to the manufacturer’s recommendation, mRNA was fragmented at 94°C for 8 minutes. Libraries were then prepared according to the manufacturer’s recommendations. Adapter concentrations and number of amplification cycles were adjusted regarding the amount of starting material. Library quantity was measured using Qubit HS DNA assay kit and quality was assessed using a Bioanalyzer high sensitivity kit on an Agilent 2100 Bioanalyzer (Agilent 5067–4626). The libraries were submitted for sequencing at Cancer Research UK Cambridge Institute Genomic Core Facility using Illumina NovaSeq with 150bp single-end reads.

### Quantitative PCR

At 24 hours post injection with mineral oil containing wasp homogenate, RNA was extracted from ten groups of 8 whole larvae, along with the same number of extractions from uninfected control larvae for each species (*D. simulans*, *D. sechellia* and F1 hybrids). RNA was isolated using RNeasy plus mini columns (Qiagen 74134). The larvae were homogenised in 400 μL of RTL plus buffer supplemented with 4 μL b-mercaptoethanol by crushing with a pestle, then immediately frozen in liquid nitrogen and stored at -70°C. Samples were thawed and centrifuged at maximum speed in a micro-centrifuge for 3 minutes to pellet debris. 350 μL of supernatant was removed to a gDNA eliminator column and RNA was isolated according to the manufacturer’s recommendation, including DNAase treatment. RNA was eluted in 30 μL of nuclease free water.

cDNA was synthesised as follows: for each sample, 1 μL of RNA was added to 0.5 μL of 10 mM of each dNTP, 0.5 μL of 0.5 μg/μL OligodT primer and 4.75 μL of nuclease free water. Samples were incubated at 65°C for 5 minutes and snap frozen on ice for 5 minutes. Next a 4.25 μL master mix containing 2 μL of 5x Superscript IV buffer (Thermofisher #10890010) 0.5 μL DTT, 0.25 μL RNasin plus (Promega N2611) and 0.5 μL Superscript IV. Samples were incubated at 50°C for 10 minutes and then heat inactivated by incubating at 80°C for 10 minutes. cDNA samples were diluted by adding 90 μL of nuclease free water.

The longest conserved constitutive exons (see below) of selected genes from *D simulans* Sz49 and *D sechellia* DenisNF100 were used for primer design with Primer-BLAST [58]. qPCR primers were designed so that amplicons contained at least one SNP that differentiated the two species, and the primers contained no SNPs. Each primer pair was tested using a 5x dilution series using hybrid gDNA as a template. Selected primers displayed efficiencies of 100% + /− 5% and single peaks on their melt-curves. qPCR was carried out using SensiFAST Hi-Rox SYBR reagents (Meridian BIO-92020) on a Step-One plus qPCR cycler (Applied Biosystems). Each cDNA was assayed for gene expression using 2 technical replicates in reactions containing 5 μL Sybr, 2 μL nuclease free water, 1 μL of 2.5 μM each primer and 2 μL of diluted cDNA with a fast PCR protocol: 95°C for 2 minutes then 40 amplification cycles of 95°C for 5s, 60°C for 30s followed by a melt curve program consisting of 95°C for 15s then 15s each at 0.3°C increments between 60°C and 95°C. The gene of interest expression was taken from the cycle threshold (*Ct*), and was normalised with that of the house-keeping gene *Rpl32* as Δ*Ct*_*gene*_ = *Ct*_*Rpl*32_−*Ct*_*gene*_.

### Amplicon sequencing

To measure allele-specific expression in specific genes, we created F1 hybrids and amplified the target transcripts using the same primers we used for quantitative PCR. For each hybrid cDNA sample, amplicons from 13 different genes were amplified in 15 μL reactions containing 7.5 μL Q5 High-Fidelity 2X Master Mix (New England Biolabs M0492S), 2 μL of template cDNA, 33 nM each primer and 4 μL of nuclease free water. Primers were designed to incorporate 5’ adapter sequences for the subsequent indexing PCR (see S1 Table for primers). Amplicons were identical to those used for qPCR. To test for PCR biases that favoured the allele of one species over the other species, we included genomic DNA from F1 hybrids as a control. To check that we could assign reads correctly to each species, we included a control of cDNA from the parental *D. sechellia* and *D. simulans* lines. A touch-down PCR program was used comprising 95°C for 3 minutes, 10 cycles of 95°C for 30s, 62°C for 15s decreasing by 1°C every cycle, 72°C for 30s, then 25 cycles of 95°C for 30s, 52°C for 15s, 72°C for 30s. Amplicons for all 13 genes were pooled together using equal volumes of PCR product. 30 μL of each pool was purified by using 3x volumes of ProNex beads (Promega NG2001) and processing according to the manufacturer’s recommendations. The purified pools were eluted from the beads by adding 30 μL of elution buffer (10 mM Tris, pH8.0). To add indexes to each pool a further PCR was carried out containing 12.5 μL Q5 High-Fidelity 2X Master Mix, 2.5 μL of Nextera index 1, 2.5 μL of Nextera index 2 (Illumina FC-131–1001), 2.5 μL of purified pool and 5 μL of nuclease free water. The PCR program was 95°C for 3 minutes, 8 cycles of 95°C for 30s, 55°C for 30s, 72°C for 30s, then 72°C for 5 minutes. Indexed libraries were pooled together using equal quantities of PCR products, and 30 μL was taken and purified using 90 μL of ProNex beads as before. The multiplex was eluted from the beads in 30 μL of 2 mM Tris pH8.0 and quantified using Qubit DNA high sensitivity kit (Thermofisher Q32854). The mean fragment size of the multiplex was 220bp, it was diluted to 10 nM with 10 mM Tris pH8.0 and submitted for sequencing at the University of Cambridge, Department of Biochemistry DNA sequencing facility using Illumina MiSeq with 300bp paired end reads.

### Digital cytometry

The digital cytometry method CIBERSORTx [32] was used to deconvolute the bulk RNA-seq data and infer hemocyte proportions in the parent *Drosophila* species and the hybrids using the reference single cell profile generated for *D. melanogaster* by Leitão et al. [31]. The top 2000 genes that had highly variable expression among cell types in that study and genes that had an average log_2_ expression greater than or equal to 0.05 across cell types were used to generate a signature matrix profile for the deconvolution. The following default recommended parameters were used for generating the signature matrix profile: a) no quantile normalisation was performed, b) a *κ* of 999 was used, c) the expression levels of 300–500 genes were used for barcoding cell types, d) a q-value threshold of 0.01 was used to test for differential expression, e) only cell phenotypes that were replicated a minimum of five times were retained and f) half of the available gene expression profiles were randomly sampled to generate the profile. After this, the cell fractions in the bulk RNA-seq were imputed using S-mode batch correction. 100 permutations were used to test the significance of cluster inferences.

### Analysis of transcription factor binding sites in fat body immune responsive genes

The FIMO tool [59] in the MEME suite [60] was used for searching the 1-kb upstream regions of coding sequences in *D. simulans* and *D. sechellia* genomes for transcription factor binding site (TFBS) motifs. We searched for TFBS motifs of 6 immune related transcription factors (TF): Relish [37, 61], Dif [37, 62, 63], STAT [64], Serpent [65], Dorsal [65] and CrebA [66] (see S7 Dataset for motif sequences). For each TF, the 531 fat body immune responsive genes were categorised as motif present (motif/+) or motif absent (motif/-) in both *D. simulans* or *D. sechellia*. The *trans*-divrged immune responsive genes were also categorised as *D. simulans* or *D. sechellia* biased in their divergence according to the log_2_ fold change estimated from the allele specific expression analysis. For each TF, a *χ*^2^ test was carried out to test if *trans*-divergence was associated with the presence of TFBS motifs (we required the motifs to be present in both *D. simulans* and *D. sechellia*) (S2A and S2B Table). Another *χ*^2^ test was carried out to test if *cis*-divergence was associated with genes where TFBS motifs were found in only one of the two species (S2C Table).

### Generating alternative genomes for *D. simulans* and *D. sechellia*

We used publicly available data to produce reference genomes for the *Drosophila* lines we were using. Whole genome sequencing data for Sz49 was obtained from NCBI Sequence Read Archive, under BioProject PRJNA318623 (BioSample: SAMN05157406) [54]. Whole genome sequencing data for DenisNF100 was obtained from NCBI Sequence Read Archive, under BioProject PRJNA395473 (BioSample: SAMN07407394) [56].

Genomic sequencing reads of Sz49 and DenisNF100 were mapped to *D. simulans* reference genome (FlyBase Dsim_r2.01) [67, 68] and the *D. sechellia* reference genome (FlyBase Dsec_r1.3) [67] respectively using the default parameters of the BWA-MEM algorithm in the BWA package [69]. Duplicated read pairs were removed using Markduplicates in the Picard toolkit (http://broadinstitute.github.io/picard/). Subsequently, variants were called against the reference genome using the GATK (v4.1.4.1) [70] HaplotypeCaller toolkit. Single nucleotide polymorphisms (SNPs) and indels were separated for Base Quality Score Recalibrations (BQSR) [70]. Each round of BQSR (GATK toolkit) was performed using hard-filtered variants from the previous round as “true set”. For SNPs, the hard-filtering criteria were set with QualByDepth < 2.0, StrandOddRatio>3.0, FisherStrand > 60.0, RMSMappingQuality<40.0, MappingQualityRankSumTest < -12.5 and ReadPosRankSumTest < -8.0. For indels, the hard-filtering criteria were set with QualByDepth < 2.0, FisherStrand > 200.0, ReadPosRankSumTest < -20.0. BQSR were performed until the number of output variants plateaued or oscillated around a constant number. The finalised sets of variants after BQSR were then used to modify the reference genomes and generate Sz49 and DenisNF100 genomes with GATK FastaAlternateReferenceMaker toolkit.

### Obtaining allele-specific expression estimates

Raw RNA sequencing reads were trimmed using Trim Galore (http://www.bioinformatics.babraham.ac.uk/projects/trim_galore/), setting the low-quality Phred score at 30 for quality control and using the first 13 base pairs from Illumina adaptors for adaptor trimming. Low-quality bases were trimmed from the 3’ end, and reads with fewer than 50 bases remaining were removed.

To remove ambiguity from allele-specific expression data, only the reads that uniquely mapped with no mismatches to the genomes of one species while not mapping to the other species are considered allele-specific reads and included in the subsequent analyses. To make our analysis robust to errors in the Sz49 and DenisNF100 genomes we created, the published reference genomes Dsim_r2.01 and Dsec_r1.3 were also incorporated in the mapping pipeline (S1 Fig). For instance, to obtain RNA sequencing reads from the *D. simulans* allele, reads were first mapped to our *D. sechellia*DenisNF100 genome followed by the Dsec_r1.3 reference genome to identify non-*D. sechellia* reads. These reads were then collected and mapped to our Sz49 genome, followed by the Dsim_r2.01 reference genome, *D. simulans*. The reads which mapped to either of these genomes were considered to be from the *D. simulans* allele. The same procedures were repeated to identify *D. sechellia* RNA reads for each library. Note that this procedure means that any read that maps to both species is discarded and not included in any of our analyses. As a consequence, our read counts do not reflect absolute expression levels of a gene. Read mapping was performed using bowtie [71] with settings allowing 0 mismatches and up to 3 reportable alignments. Only the reads that had a single reported alignment were retained for analysis.

Expression was represented by the number of reads mapped to the gene in question. To ensure we are studying differences in expression level rather than patterns of alternative splicing, we only considered exons that were present in all the transcripts produced by a gene (‘constitutive exons’). To identify the location of homologous exons in the two species, we needed to convert the genome coordinates of an exon in one species to the genome coordinates of the other species. Furthermore, to prevent changes in gene length being mistaken for changes in expression, we only included exon sequence that was conserved between *D. simulans* and *D. sechellia* (‘conserved exons’). Exons only present in one species were not considered, and where the exon boundaries had changed, we altered the exon boundaries so that they were the same in both species. The approach we took was to convert the genomic coordinates of each read mapped to the constitutive exons of one species to the coordinates of the other species, and we discarded the read if it did not overlap with an exon in that species. This genomic coordinates conversion was done using the LiftOver tool developed by UCSC [72]. The chain files for converting Dsim_r2.01 and Dsec_r1.3 coordinates were provided by UCSC Genomic Institute. The chain files for Sz49 to Dsim_r2.01 and DenisNF100 to Dsec_r1.3 coordinates conversion were generated locally with UCSC command line tools. Read counts were generated with HTSeq-count [73]. The procedures of annotating allele-specific gene expressions are detailed in S3 Fig. This pipeline is designed to accommodate the available chain files and published annotation files for Dsim_r2.01 and Dsec_r1.3.

### Statistical analyses of RNAseq data

Low expression genes that had a count per million (CPM) < = 3 in at least 3 hemocytes libraries or at least 5 fat body libraries were filtered out. A CPM of 3 is equivalent to 10 mapped reads in the smallest hemocytes library and 18 mapped reads in the smallest fat body library. The R package edgeR [74] was used for differential expression analyses with RNAseq data. Dispersions were measured using the Cox-Reid profile adjusted likelihood method before fitting the expression data with a negative binomial generalised linear model. Differential expression was determined using a quasi-likelihood *F*-test, and a false discovery rate (FDR) of 0.05 was set as the significance threshold.

To compare expression levels, we used a generalized linear model (GLM) implemented in edgeR [74]. This allows us to perform different contrasts both to estimate *P* values and fold changes in gene expression. First, to examine the expression patterns when flies mount an immune response, we analysed each species and F1 hybrids separately, and the contrast was set between the immune challenged and control samples. Second, to identify genes that were deferentially expressed between *D. simulans* and *D. sechellia*, we analysed the immune challenged and control groups separately, and the contrast was set between species. Third, we examined how the immune response had diverged between species. To achieve this, we contrasted the transcriptional response to immune challenge in *D. simulans* to the transcriptional response in *D. sechellia*.

These comparisons between species detect change in gene expression caused by both (*cis* and *trans* factors. To investigate change in *cis* alone, we looked for differences in the expression of the two alleles in F1 hybrids. To detect allele-specific differential expression, we repeated the contrasts between species, but this time the data was read counts from the two alleles in the F1 hybrids.

Having analysed divergence between the species (*cis* and *trans*) and allele-specific expression (*cis*), we could then use the difference between these to estimate divergence in *trans*. Again, the magnitude and significance of *trans* effects was estimated by performing this contrast within the GLM framework of edgeR.

We placed genes within different categories of expression divergence following McManus, 2010 [14]. *Cis*: significant ASE within F1 hybrids, no significant *trans* divergence; *Trans*: significant *trans* divergence but no significant ASE within F1 hybrids; *Cis* + *Trans*: ASE within F1 hybrids, divergence between parents and divergence in *trans* are all significant, with *cis* and *trans* divergence in the same direction; *Cis* x *Trans*: ASE within F1 hybrids, divergence between parents and divergence in *trans* are all significant, with *cis* and *trans* divergence in the opposite direction; Compensatory: significant ASE within F1 hybrids and in *trans*, but no significant divergence between parents; Conserved: no significant change in gene expression in any of the comparisons; Ambiguous: all the rest. We noticed that many ambiguous genes resulted from our arbitrary choice of significance threshold – the divergence between species crossed the threshold, but *cis* and *trans* effects did not. Among the ambiguous genes, we therefore examined the magnitude of the *cis* and *trans* log fold change estimates. If the cis or trans effect explained at least half the divergence of the two species, we classified the gene as ‘*cis*’ or ‘*trans*’. *Cis* diverged genes include those categorised as *Cis*, *Cis* + *Trans*, *Cis* x *Trans* and Compensatory. *Trans* diverged genes include those categorised as *Trans*, *Cis* + *Trans*, *Cis* x *Trans* and Compensatory. Both *Cis* and *Trans* diverged genes include those categorised as *Cis* + *Trans*, *Cis* x *Trans* and Compensatory.

Gene ontology enrichment analysis was performed using FlyMine [75] database and tools, with the 4969 analysed fat boy genes as background population.

### Accuracy of estimates of allele-specific expression

To investigate the relative contribution of *cis-* and *trans-* regulatory changes to the expression divergence between *D. simulans* and *D. sechellia*, we compared the expression of the two alleles in F1 hybrids (allele-specific expression). To do this, we assigned RNAseq reads to *D. simulans* and *D. sechellia* using sequence differences between the species (S1 and S3 Figs). As the two alleles share the same *trans-*regulatory environment in hybrids, allele-specific expression is a measure of *cis-* regulatory divergence between the species [26]. Expression differences between the parental species is caused by both *cis* and *trans* divergence, so *trans* effects can be estimated by subtracting the *cis* effects from the total divergence between parents [10].

We used two approaches to confirm we could accurately assign RNAseq reads to the correct species. First, because all the data went through the same analysis pipelines (S1 and S3 Figs), we can estimate our error rate by checking whether reads from the parental species were always assigned to that species. This allowed us to filter out genes with a > 0.01 mismapping rate in either parental species. After also removing genes with low expression levels, there were 4969 and 5275 genes remaining in fat body and hemocyte data-sets respectively. In this dataset, we could successfully allocate the large majority of reads to the correct species, although more mismapping happened to reads from *D. simulans* (S2(A) Fig), likely due to higher genetic diversity in this species. Two hemocytes libraries (B7 and D7) had a low level of contamination between species (S2(A) and S2(C) Fig). Secondly, we compared the total number of reads from the two species in F1 hybrids. In these libraries, read counts from *D. simulans* and *D. sechellia* are very similar, again suggesting no systematic bias in assigning reads to species (S2(B) and S2(C) Fig).

### Quantifying allele-specific expression using amplicon sequencing

Following amplicon sequencing, Illumina MiSeq reads of the 13 selected genes were mapped to the *D. simulans* (Sz49) genome sequence using the default parameters of the BWA-MEM algorithm in the BWA package [69]. The amplicons had been selected so that they contained SNPs that allowed us to distinguish reads from the *D. simulans* and *D. sechellia* alleles. We checked that these were homozygous in the parental species by visual inspection in Integrative Genomics Viewer (IGV_2.9.4) [76].

To estimate the relative number of transcripts from the *D. simulans* and *D. sechellia* alleles in F1 hybrids, we counted the number of reads with each SNP allele using tools implemented in Rsamtools [77]. For genes with more than one SNP in the amplicon, we confirmed that the different SNPs resulted in similar estimates of allele-specific expression. As this was the case, we only analysed data from the first SNP from the 5’ end. We tested if there was a bias towards the *D. simulans* or *D. sechellia* alleles by analysing reads generated by amplifying genomic DNA from F1 hybrids, and checking half the reads came from each species.

Transcript counts for each gene were analysed by fitting a mixed effects logistic regression using the *glmer* function in the R package lme4 [78]. We treated allele (*D. simulans*/*D. sechellia*) and treatment (control/infected) as fixed effects and sample identity as a random effect (i.e., this random effect estimated the residual variance and therefore accounts for over-dispersion). As a measure of the extent of *cis* divergence, we estimated the log odds (logit) of an RNAseq read being derived from the *D. simulans* allele under either controlled or infected conditions. This parameter was extracted from estimated coefficients in the regression model. A logit equal to zero suggest no difference between the expression of the two species’ alleles in hybrids, and therefore no interspecific *cis-*regulatory divergence.

### Statistical analysis of qPCR data

Expression levels of the 13 selected genes were obtained from qPCR cycle thresholds (*CT*) normalised using the reference gene *Rpl32* (Δ*CT*). In the F1 hybrids, we then multiplied these Δ*CT* values by the proportion of transcripts from the *D. simulans* and *D. sechellia* alleles (allele-specific expression, estimated as described in the previous section), giving us the expression of each allele separately. The Δ*CT* values in the parental species were divided by two to make them comparable to the expression levels in hybrids.

To estimate *trans-*regulatory divergence in these genes, we assessed the goodness of fit of a full model fitted with relative expression levels against that of a series of its nested models using likelihood ratio test implemented by R package lmtest [79]. For the full model, the allelic expression levels of each gene (*CT* values from PCR programs) from both parental and hybrid libraries were fitted into a general linear regression with ‘lm’ function in R package stats [80], with allelic type (*D. simulans*/*D. sechellia*), species (parents/hybrids), treatment (controlled/infected) and their interactions (allelic type:species, allelic type:treatment, species:treatment, allelic type:species:treatment) as parameters. We compared the full model with all 7 parameters with the nested model with a subset of parameters (allelic type, treatment, allelic type:treatment) excluding terms concerning “species” to evaluate the interspecific divergence of *trans-*effect on relative gene expressions, i.e., differential expression patterns between parental alleles and hybrid alleles. The *trans* contribution to interspecific divergence under controlled or infected conditions was estimated from the coefficient measuring “allele type” and “species” interaction (species:allele type) in the full regression model, i.e., the relative abundance between allele transcripts depending on whether in parental species or hybrids. The controlled and infected *trans* divergences were inferred from the full model by setting the intercept at “control” and “wasp” status, respectively. The effect of *trans-*regulatory divergence on immune response was represented by the three-way interaction term of the parameters (allele type:species:treatment) in the full regression model. It is the difference of *trans* contribution in interspecies divergence between control and infected status.

## Supporting information

S1 Dataset RNAseq read counts for immune responsive genes in hemocytes. The column titles include the type of sample in upper case (*D. sechellia, D. simulans* or hybrid), and the allele the reads were assigned to in lower case (*D. sechellia* or *D. simulans*).
(CSV)

S2 Dataset RNAseq read counts for immune responsive genes in the fat body. The column titles include the type of sample in upper case (*D. sechellia, D. simulans* or hybrid), and the allele the reads were assigned to in lower case (*D. sechellia* or *D. simulans*).
(CSV)

S3 Dataset Differential expression of genes in response to immune challenge within each species. This data was used to plot Figs 2A, 2B and 3B.
(CSV)

S4 Dataset Proportion of lamellocytes and their precursors inferred using digital cytometry. This data was used to plot Fig 3A.
(CSV)

S5 Dataset Differential expression of genes in cis and trans in hemocytes. This data was used to plot Fig 4.
(CSV)

S6 Dataset Differential expression of genes in cis and trans in hemocytes. This data was used to plot Fig 5.
(CSV)

S7 Dataset Transcription factor binding motifs. (TXT)

S8 Dataset Read counts from amplicon sequencing of selected immunity genes. This data underlies Figs 6 and 7.
(CSV)

S9 Dataset Gene expression of selected genes estimated by quantitative PCR. The data is raw cycle threshold values, and was combined with S8 Dataset to generate Fig 7.
(XLS)

S10 Dataset Read counts per million (cpm) for immune responsive genes in the fat body. Separate cpm values are given for each library. This data underlies Fig 3B, and was combined with the statistical significance values in S3 Dataset to generate Fig 3B.
(CSV)

S11 Dataset Differential expression of genes between *D. simulans* and *D. sechellia*. This data was used to generate Fig 5.
(CSV)

S1 Table Primer sequences used for both quantitative PCR and amplicon sequencing. (PDF)

S2 Table Number of *cis* or *trans* diverged immune responsive genes in the fat body, with or without transcription factor binding site motifs. Immune-responsive genes in the fat body were classified as to whether they had motifs associated with the binding of six transcription factors. In (A) and (B) Motif/+ genes had the motif in both *D. sechellia* and *D. simulans*. In (C) Motif+ genes had the motif in just one of these species but not the other. These genes in turn were classified as to whether they had diverged in *cis* or *trans* in control or immune-challenged conditions, of if the response to infection had been changed in *trans*. The *P* value is from a chi-squared test.
(PDF)

S1 Fig Bioinformatic pipeline to obtain allele-specific RNA sequencing read counts. (PDF)

S2 Fig Accuracy of allele-specific RNA-seq read mapping. (A) The proportion of reads from single-species libraries that were incorrectly mapped to the wrong species. (B) The proportion of reads that were mapped to different species’ genomes. Each point is a different sequencing library. The colours reflect the species from which that library was made.
(PDF)

S3 Fig Obtaining allele-specific gene expressions for each library. In each library, aligned coordinates of mapped to Sz49 genome reads were converted to Dsim_r2.01 coordinates then Dsec_r1.3 coordinates with LiftOver, allowing selection for mapped to Dsec_r1.3 constitutive exons reads. The selected reads, now with Dsec_r1.3 coordinates, would be converted to Dsim_r2.01 coordinates, allowing us to see whether the read mapping to exon sequence that is found in both species (‘conserved exons’). This allowed us to use HTSeq-count to produce the read counts just those reads mapping to exon sequence present in both species. Similarly, reads mapped to Dsim_r1.2, DsecNF100 and Dsec_r1.3 genomes went through various coordinate conversions, so we could check they mapped to sequence annotated as an exon in both species’ reference genomes (Dsec_r1.3 and Dsim_r2.01). Any read that mapped to sequence annotated as an exon in only one of the reference genomes was discarded.
(PDF)

S4 Fig Dominance of gene expression in F1 hybrids. Differences in gene expression between the parental species and hybrids in hemocytes and fat body. (A) The scatter plots comparing the relative expression differences between F1 hybrids and each parental species. Each point is an immune-responsive gene (a gene that was differentially expressed after immune challenge in one or more species). The colours represent different dominance relationships. Under- and over-dominant genes have lower or higher expression in hybrids than both parental species. Additive genes have an expression level in hybrids that is intermediate between the parents. Dominant genes have expression levels that resemble one parent. Following McManus 2010 [14] and Gibson 2004 [81], expression was considered different between hybrids and the parents if it was both statistically significant (FDR<0.05) and there was at least 1.25 log_2_ fold difference. (B) The number of the genes in each category.
(PDF)

S5 Fig Proportion of mature and immature lamellocytes among hemocytes. Proportions of lamellocytes on different transcriptional states in *D. simulans*, *D. sechellia* and F1 hybrid larvae under control and immune challenged conditions, estimated from bulk RNA-seq data with CIBERSORTx. The upper and lower lines on top of the bar give the 95% confidence interval of the estimate.
(PDF)
