# Supplementary material for: Trans-regulatory changes underpin the evolution of the Drosophila immune response
Source: PLoS Genet. 2022 Nov 7;18(11):e1010453. doi: 10.1371/journal.pgen.1010453 (PMC9671443; doi:10.1371/journal.pgen.1010453)
Supplement: S1 Table — (PDF) [file pgen.1010453.s012.pdf]

Gene	PrimerID	Sequence (5' to 3')	Product length (bp)	Primer efficiency (%)
BomBc1	BomBc1_p4_fw<br>BomBc1_p4_rv	CCGGATTGGACCATCGAGAG<br>GAAAACGTGGTCGTCGGTG	111	91
Indy	Indy_p7_fw<br>Indy_p7_rv	GGGGAAGTCCATCTGTTCGG<br>TCATCGGAACTGCCACCAAT	83	104
CG8850	CG8850_p6_fw<br>CG8850_p6_rv	CCCAGATAATCCAGCCAGCC<br>CCTGGTCCTCGTCTACATGC	98	99
CG16772	CG16772_p18_fw<br>CG16772_p18_rv	CCCACTTCCAAAGATGCGGA<br>TAGGGCGAGTACGGTGATCT	86	100
BomBc3	BomBc3_p5_fw<br>BomBc3_p5_rv	ATCCCCTGGCGATGGTAATG<br>CACCCCTCCGGTAAATGGTG	83	94
FBgn0196692	0196692_p15_fw<br>0196692_p15_rv	TGAGTTCCTCCAGCCGATTG<br>AGGCCCAGCTCGAGATCAT	113	102
Mtk	Mtk_p1_fw<br>Mtk_p1_rv	CGATTTTTCTGGCCCTGCTG<br>CGAAAATGGGTCCCTGGTGA	84	100
edin	edin_p10_fw<br>edin_p10_rv	AGGCCAGTGTTCAGTACAACC<br>TGCCGCCTTCGTAGTTGTT	120	95
TotX	TotX_p8_fw<br>TotX_p8_rv	GATGGTGTACCACCTCAAGGA<br>GGATTGCCCTTCCTCCCTTT	117	94
BomS1	BomS1_p2_fw<br>BomS1_p2_rv	TTCCACTGTCGCCCGATCC<br>TACTTGCCACCGTGGACATTG	76	99
CecA1	Dsim_CecA1_p1_fw<br>Dsim_CecA1_p1_rv	TCTTCGTTTTCGTCGCTCTCA<br>ATTTTCTTCAGCCAGCCAGC	70	100
CecC	Dsim_CecC_p5_fw<br>Dsim_CecC_p5_rv	CGCATTGGCCAGCACAC<br>CCACATTGGCGGCCTGTT	70	96
GD19384	GD19384_p3_fw<br>GD19384_p3_rv	CGCCCTTTCATCTGGTCTCA<br>CGAACCTCCTTGGACGACAA	99	97

Table S1. **Primer sequences used for both quantitative PCR and amplicon sequencing.**
