# Supplementary material for: Trans-regulatory changes underpin the evolution of the Drosophila immune response
Source: PLoS Genet. 2022 Nov 7;18(11):e1010453. doi: 10.1371/journal.pgen.1010453 (PMC9671443; doi:10.1371/journal.pgen.1010453)
Supplement: S2 Table — Immune-responsive genes in the fat body were classified as to whether they had motifs associated with the binding of six transcription factors. In (A) and (B) Motif/+ genes had the motif in both D. sechellia and D. simulans. In (C) Motif+ genes had the motif in just one of these species but not the other. These genes in turn were classified as to whether they had diverged in cis or trans in control or immune-challenged conditions, of if the response to infection had been changed in trans. The P value is from a chi-squared test. (PDF) [file pgen.1010453.s013.pdf]

A

	Control					Wasp					Response				
	Trans diverged *D. simulans* biased		Other			*Trans* diverged *D. simulans* biased		Other			*Trans* diverged *D. simulans* biased		Other		
Transcription factor	Motif / +	Motif / -	Motif / +	Motif / -	P value	Motif / +	Motif / -	Motif / +	Motif / -	P value	Motif / +	Motif / -	Motif / +	Motif / -	P value
Relish	6	30	63	432	0.67	6	63	44	418	1	0	69	9	453	0.50
Dif	12	149	24	346	0.83	21	29	140	341	0.08	1	8	160	362	0.37
STAT	8	28	76	419	0.39	11	39	73	408	0.29	1	8	83	439	1
Serpent	6	30	94	401	0.90	7	43	93	388	0.47	0	9	100	422	0.30
Dorsal	1	35	37	458	0.47	2	48	36	445	0.53	1	8	37	485	1
CrebA	5	31	93	402	0.61	8	42	90	391	0.78	2	7	96	426	1

B

	Control					Wasp					Response				
	Trans diverged *D. sechellia* biased		Other			*Trans* diverged *D. sechellia* biased		Other			*Trans* diverged *D. sechellia* biased		Other		
Transcription factor	Motif / +	Motif / -	Motif / +	Motif / -	P value	Motif / +	Motif / -	Motif / +	Motif / -	P value	Motif / +	Motif / -	Motif / +	Motif / -	P value
Relish	2	45	67	417	0.10	15	120	54	342	0.54	3	14	66	448	0.83
Dif	17	30	144	340	0.45	38	97	123	273	0.60	6	11	155	359	0.85
STAT	9	38	75	409	0.66	21	114	63	333	1	4	13	80	434	0.59
Serpent	12	35	88	396	0.30	23	112	77	319	0.62	3	14	97	417	1
Dorsal	4	43	34	450	0.94	11	124	27	369	0.75	2	15	36	478	0.79
CrebA	8	39	90	394	0.95	21	114	77	319	0.38	2	15	96	418	0.69

C

	Control					Wasp				
	Cis diverged		No *cis* divergence			*cis* diverged		No *cis* divergence		
Transcription factor	Motif / +	Motif / -	Motif / +	Motif / -	P value	Motif / +	Motif / -	Motif / +	Motif / -	P value
Relish	14	230	19	268	0.8108	16	238	17	260	1
Dif	18	226	20	267	0.9896	16	238	22	255	0.5719
STAT	13	231	15	272	1	13	241	15	262	1
Serpent	19	225	26	261	0.7126	19	235	26	251	0.5275
Dorsal	6	238	16	271	0.1148	8	246	14	263	0.3777
CrebA	8	236	21	266	0.0644	8	246	21	256	0.03999

Table S2. Number of *cis* or *trans* diverged immune responsive genes in the fat body, with or without transcription factor binding site motifs. Immune-responsive genes in the fat body were classified as to whether they had motifs associated with the binding of six transcription factors. In (A) and (B) Motif/+ genes had the motif in both *D. sechellia* and *D. simulans*. In (C) Motif+ genes had the motif in just one of these species but not the other. These genes in turn were classified as to whether they had diverged in *cis* or *trans* in control or immune-challenged conditions, of if the response to infection had been changed in *trans*. The *P* value is from a chi-squared test.
