# Supplementary figures and images for: Trans-regulatory changes underpin the evolution of the Drosophila immune response
Source: PLoS Genet. 2022 Nov 7;18(11):e1010453. doi: 10.1371/journal.pgen.1010453 (PMC9671443; doi:10.1371/journal.pgen.1010453)

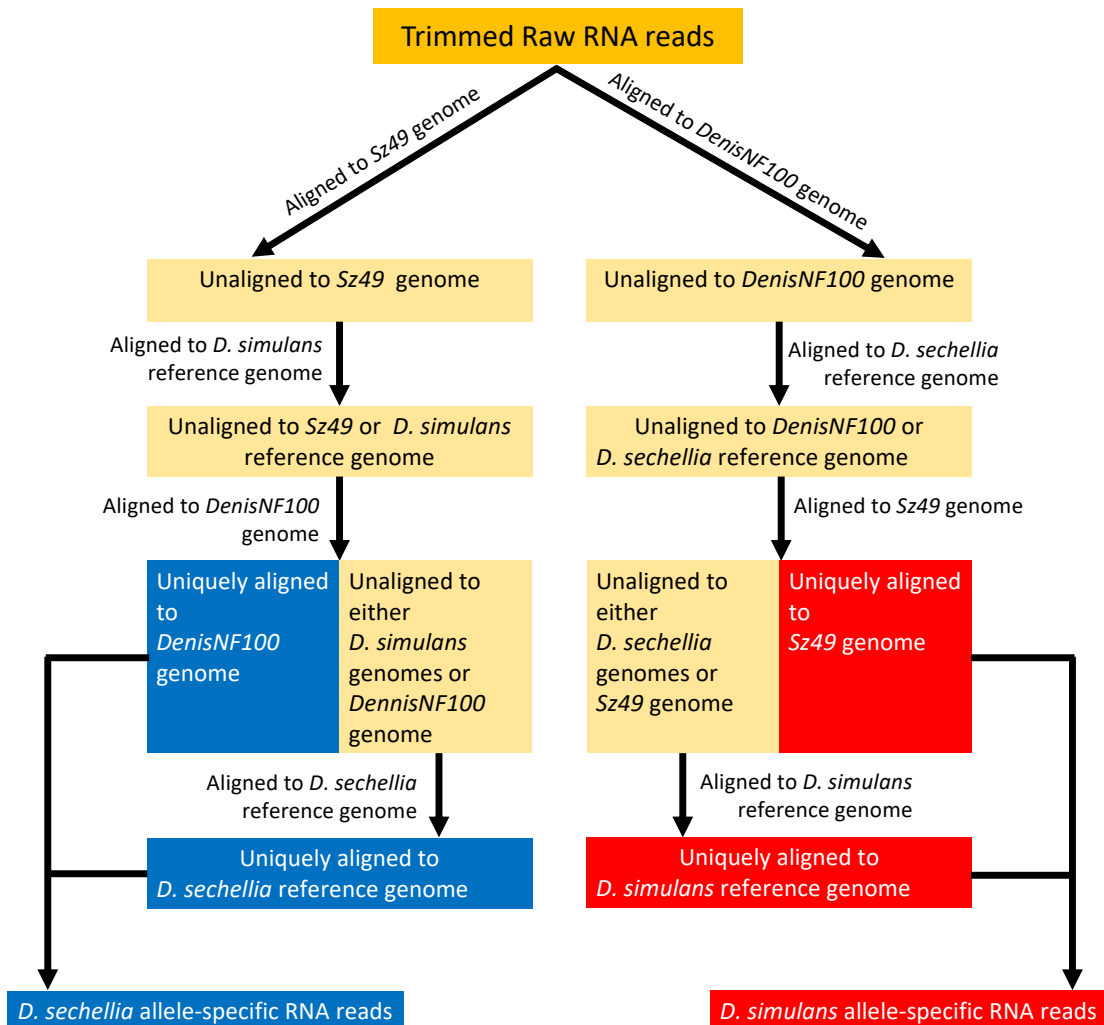

Supplement: S1 Fig — (PDF) [file pgen.1010453.s014.pdf]

A

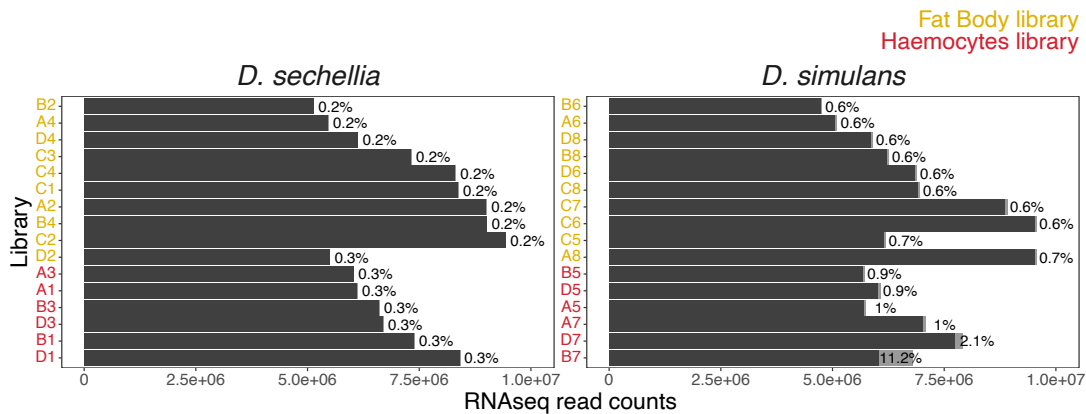

B

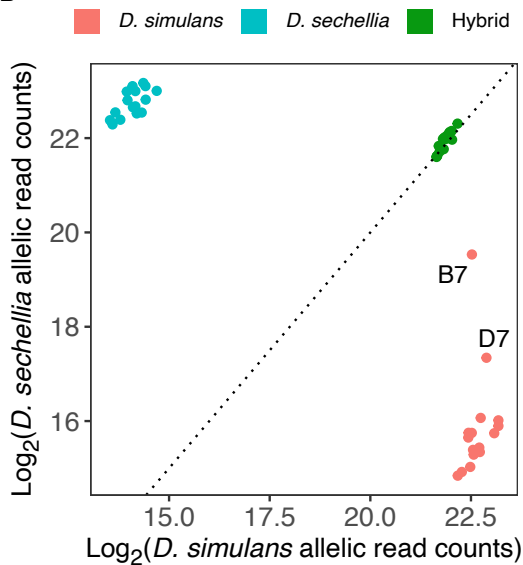

Supplement: S2 Fig — (A) The proportion of reads from single-species libraries that were incorrectly mapped to the wrong species. (B) The proportion of reads that were mapped to different species’ genomes. Each point is a different sequencing library. The colours reflect the species from which that library was made. (PDF) [file pgen.1010453.s015.pdf]

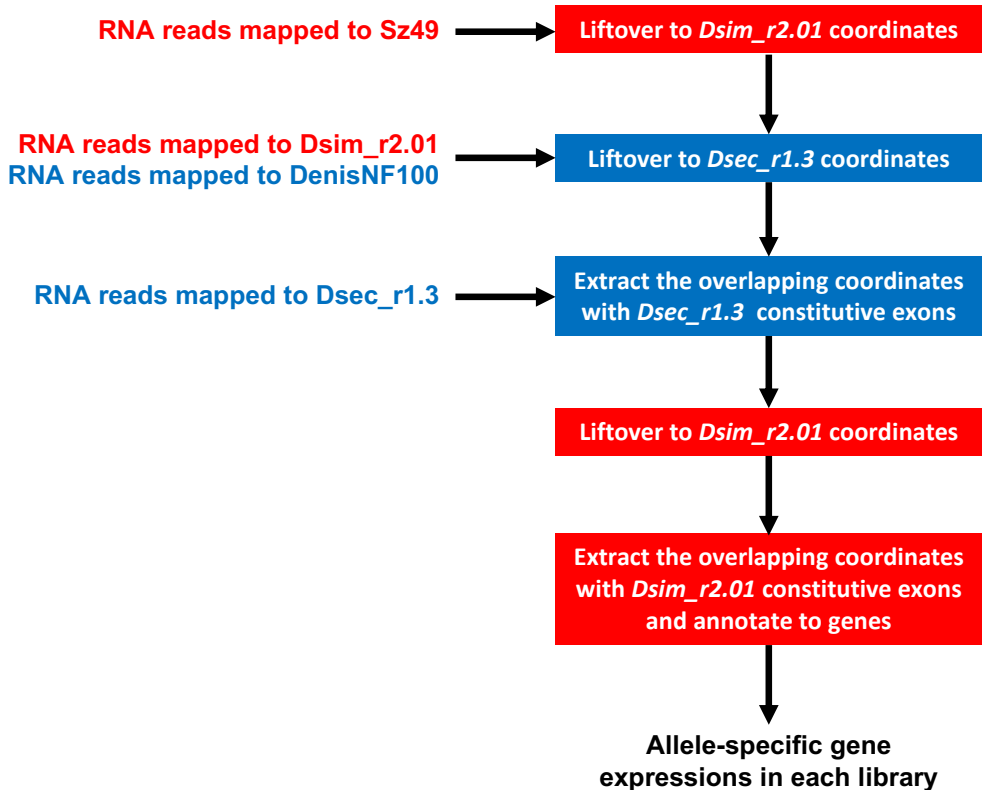

Supplement: S3 Fig — In each library, aligned coordinates of mapped to Sz49 genome reads were converted to Dsim_r2.01 coordinates then Dsec_r1.3 coordinates with LiftOver, allowing selection for mapped to Dsec_r1.3 constitutive exons reads. The selected reads, now with Dsec_r1.3 coordinates, would be converted to Dsim_r2.01 coordinates, allowing us to see whether the read mapping to exon sequence that is found in both species (‘conserved exons’). This allowed us to use HTSeq-count to produce the read counts just those reads mapping to exon sequence present in both species. Similarly, reads mapped to Dsim_r1.2, DsecNF100 and Dsec_r1.3 genomes went through various coordinate conversions, so we could check they mapped to sequence annotated as an exon in both species’ reference genomes (Dsec_r1.3 and Dsim_r2.01). Any read that mapped to sequence annotated as an exon in only one of the reference genomes was discarded. (PDF) [file pgen.1010453.s016.pdf]

A

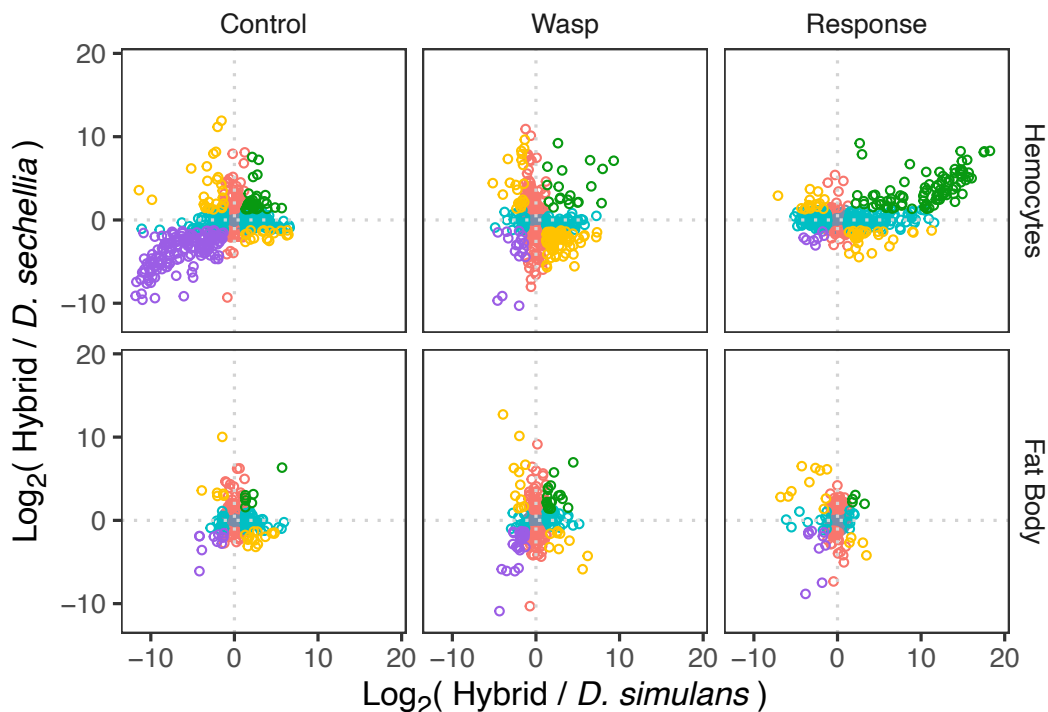

B

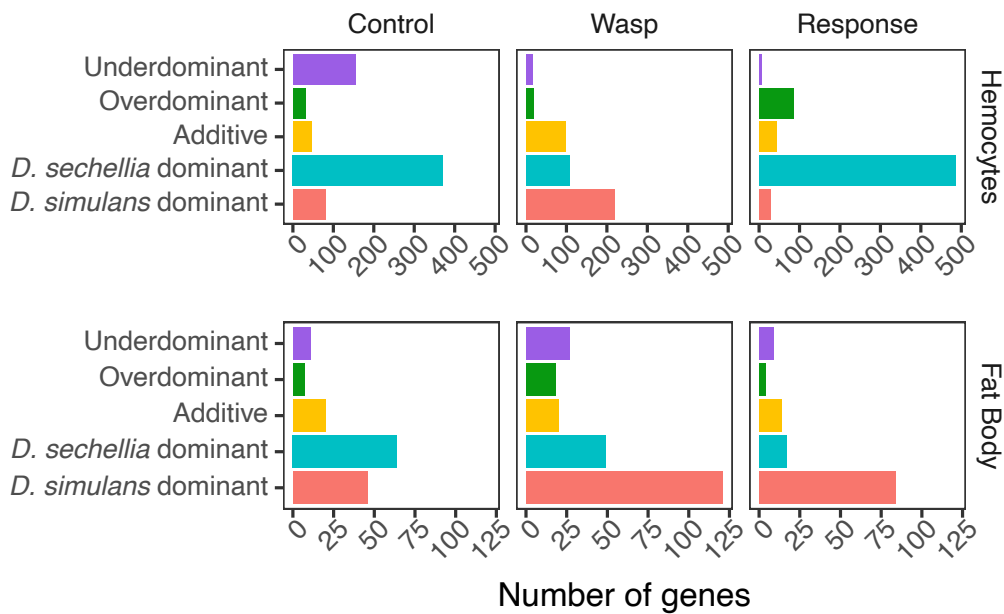

Supplement: S4 Fig — Differences in gene expression between the parental species and hybrids in hemocytes and fat body. (A) The scatter plots comparing the relative expression differences between F1 hybrids and each parental species. Each point is an immune-responsive gene (a gene that was differentially expressed after immune challenge in one or more species). The colours represent different dominance relationships. Under- and over-dominant genes have lower or higher expression in hybrids than both parental species. Additive genes have an expression level in hybrids that is intermediate between the parents. Dominant genes have expression levels that resemble one parent. Following McManus 2010 [14] and Gibson 2004 [81], expression was considered different between hybrids and the parents if it was both statistically significant (FDR<0.05) and there was at least 1.25 log2 fold difference. (B) The number of the genes in each category. (PDF) [file pgen.1010453.s017.pdf]

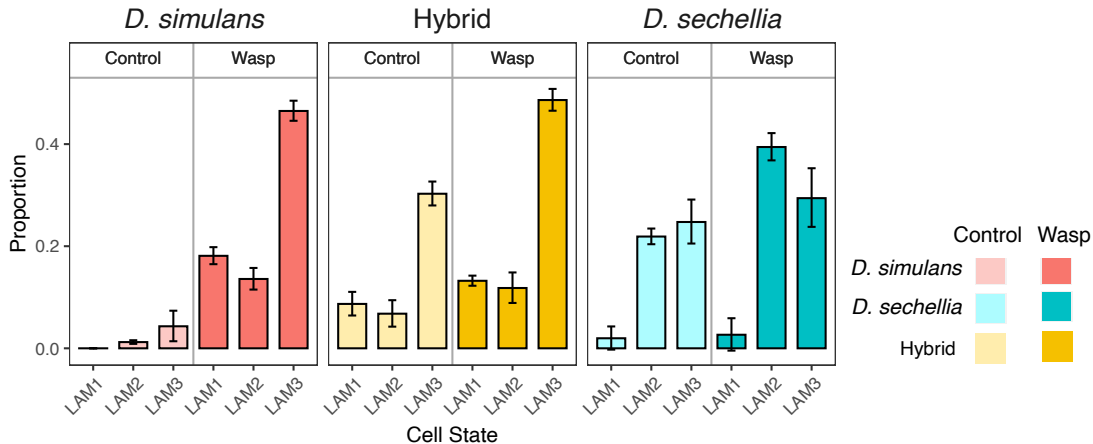

Supplement: S5 Fig — Proportions of lamellocytes on different transcriptional states in D. simulans, D. sechellia and F1 hybrid larvae under control and immune challenged conditions, estimated from bulk RNA-seq data with CIBERSORTx. The upper and lower lines on top of the bar give the 95% confidence interval of the estimate. (PDF) [file pgen.1010453.s018.pdf]
